# Effect of copper and nickel exposure on ribosomal DNA variation in *Daphnia pulex* mutation accumulation lines

**DOI:** 10.1093/g3journal/jkae305

**Published:** 2024-12-23

**Authors:** Abir Elguweidi, Melania E Cristescu, Teresa J Crease

**Affiliations:** Department of Integrative Biology, University of Guelph, Guelph, Ontario, Canada N1G 2W1; Department of Animal Science, University of Benghazi, Benghazi, Libya 21861, North Africa; Department of Biology, McGill University, Montreal, Quebec, Canada, H3A 1B1; Department of Integrative Biology, University of Guelph, Guelph, Ontario, Canada N1G 2W1

**Keywords:** mutation accumulation lines, *Daphnia pulex*, ribosomal DNA, copy number variation, metal effects

## Abstract

The release of heavy metals from industrial, agricultural, and mining activities poses significant risks to aquatic ecosystems by degrading water quality and generating reactive oxygen species that can damage DNA in aquatic organisms. *Daphnia* is a widespread keystone species in freshwater ecosystems that is routinely exposed to a range of anthropogenic and natural stressors. With a fully sequenced genome, a well-understood life history and ecology, and an extensive library of responses to toxicity, *Daphnia* serves as an ideal model organism for studying the impact of environmental stressors on genomic stability. Ribosomal DNA (rDNA) encodes ribosomal RNA (rRNA), which is essential for protein synthesis, and the spacers that separate the rRNA genes contain regulatory elements. However, the effects of heavy metals on this region of the genome are not well documented. We used short-read whole-genome sequences to analyze copy number and sequence variation in *Daphnia pulex* mutation accumulation lines exposed to nickel and copper, both individually and in combination, at concentrations that mimic levels often found at contaminated sites. We found no significant direct effect of chronic exposure to either metal on rDNA copy number or sequence variation. However, the results suggest that nickel and copper exposure may indirectly influence rDNA by altering recombination rates. We also emphasize the importance of interval length between generational samples for accurately assessing the frequency and magnitude of rDNA copy number changes. In addition, we observed differential expansion of rDNA haplotypes, suggesting that they may be clustered within the rDNA array.

## Introduction

Human activities such as mining and intensive agriculture have caused significant heavy metal pollution in aquatic ecosystems (Baby *et al*. 2010). This contamination poses a severe threat to the health and survival of aquatic organisms due to its persistence and ability to bioaccumulate (Kim *et al*. 2017). Exposure of aquatic invertebrates to heavy metals is associated with increased production of reactive oxygen species (ROS), depletion of glutathione, inhibition of oxidative phosphorylation and antioxidant systems, DNA damage, and impaired DNA repair mechanisms (Chiarelli and Roccheri 2014). Additionally, heavy metals modulate the expression of genes involved in protecting cells from metal-induced oxidative stress (Kim *et al*. 2017), which can inhibit some DNA repair mechanisms and disrupt cell proliferation (Lloyd and Phillips 1999).

Mining and smelting activities in Sudbury, Ontario, Canada, have historically led to acidification and elevated metal contamination, especially nickel (Ni) and copper (Cu), in thousands of lakes (Keller *et al*. 2007). While water quality has improved significantly in recent decades, Ni and Cu levels still often exceed Canadian water quality guidelines. Consequently, key zooplankton species, such as *Daphnia*, have had difficulty recovering, likely due to the persistent presence of metal pollutants (Labaj *et al*. 2015; Celis-Salgado *et al.* 2016). Taylor *et al*. (2016) investigated how Ni and Cu affected *Daphnia pulex–Daphnia pulicaria* hybrids from Sudbury lakes. They found that exposure to both metals, individually and in combination, led to significant reductions in reproductive output. These effects were particularly pronounced at low concentrations, likely due to the soft water and low pH. Specifically, Cu exposure decreased reproduction by 9–30%, while Ni exposure reduced it by 21–27%. Combined Ni and Cu exposure led to even greater reproductive impairment. In addition, Cu exposure disrupts immunity and metal regulation in *D. pulex* while enhancing the expression of genes related to growth and digestion. Cu-tolerant lineages better regulate stress genes like metallothionein, suggesting adaptive advantages conferred by prior exposure. This indicates that historical exposure shapes gene expression, improving Cu tolerance (Chain, Finlayson, *et al*. 2019). Furthermore, Liorti *et al*. (2017) found that calcium reduces Cu toxicity in juvenile *D. pulex*, with survival highest at low Cu/high Ca and lowest at high Cu/low Ca levels. Acute Cu exposure in *D. pulex* triggers unique molecular responses, including differential gene expression and alternative splicing with genetic background having more pronounced effect on alternative splicing than on gene expression (Suresh *et al*. 2020). Moreover, Cu exposure induces DNA methylation changes in *D. pulex*, potentially inherited across generations. These changes influence gene regulation through epigenetic mechanisms (Jeremias *et al*. 2022).

Ribosomal DNA (rDNA) is a multigene family organized in tandem arrays on chromosomes. The rDNA repeat unit in eukaryotes typically contains coding regions for 5.8S, 18S, and 28S ribosomal RNA (rRNA), which are separated by internal transcribed spacers (ITS1 and ITS2). Each transcribed unit is further separated by an intergenic spacer (IGS), which includes the external transcribed spacer and a nontranscribed spacer, both containing regulatory and subrepeat elements (Long and Dawid 1980). rDNA undergoes concerted evolution, which is primarily driven by unequal recombination between repeats. This recombination can cause changes in rDNA copy number and leads to higher homogeneity among rDNA units within species than between species (Eickbush and Eickbush 2007).

The function of rDNA extends beyond ribosome biogenesis as it also plays a key role in the regulation of gene expression (Paredes *et al*. 2011; Larson *et al*. 2012; Gibbons *et al*. 2014). Changes to rDNA can influence chromatin states and are important for epigenetic regulation (Paredes and Maggert 2009). Several studies have observed a reduction in rDNA copy number during tumor progression, suggesting a link between rDNA copy number variation (CNV) and tumorigenesis (Xu *et al*. 2017; Wang *et al*. 2017; Lou *et al*. 2021).

Copy number changes in rDNA can occur rapidly within a few generations, even though the average remains consistent between parents and offspring (Rabanal *et al*. 2017; Kindelay and Maggert 2023; Salim and Gerton 2019). Such changes can significantly affect genome size, potentially making it a target of selection, as observed in *Arabidopsis thaliana*, which has a compact genome (Long *et al*. 2013). Additionally, the size of the rDNA array may influence nonribosomal functions, such as the maintenance of genome integrity (Kobayashi *et al*. 2004).

Several studies have investigated the impact of environmental stressors on rDNA. For example, Waters and Schaal (1996) found that a 2-h heat shock at 40°C affected genomic components of *Brassica nigra*, reducing the number of rDNA copies passed to the next generation, possibly due to DNA damage and repair mechanisms triggered by the heat. Similarly, Ide *et al*. (2010) found that UV radiation and the chemical mutagen methyl methanesulfonate increased rDNA recombination rates. A recent study of *D. pulex* suggested that Ni and Cu exposure could cause fluctuations in rDNA copy number by disrupting rDNA regulation, potentially activating mechanisms such as extrachromosomal circle formation (Harvey *et al*. 2020). However, few studies have specifically examined the effects of heavy metals on rDNA.

Research on *Daphnia* has revealed variation in rDNA copy number and sequence polymorphism. For example, studies using restriction site analysis (Crease and Lynch 1991) and sequencing of cloned rDNA repeats from individuals of the *D. pulex* species complex (Crease 1995; McTaggart and Crease 2005; Ambrose and Crease 2011) identified intraindividual sequence variation. Further work using quantitative PCR (qPCR) detected rDNA CNV in *D. pulex* (Eagle and Crease 2012, 2016) and *Daphnia obtusa* (LeRiche *et al*. 2014). More recently, Elguweidi and Crease (2024) reported significant variation in rDNA copy number and sequence across 10 natural populations of *D. pulex* using short-read genome sequences.

*Daphnia*, a freshwater zooplankter in the crustacean order Cladocera, is extensively used in ecological, developmental, and ecotoxicological research, making it an important model organism for studies in these fields (Ebert 2005; Reynolds 2011). *Daphnia* plays a vital role in aquatic ecosystems by grazing on phytoplankton and serving as a key prey species for fish and other predators (Miner *et al*. 2012). *Daphnia* is routinely exposed to a variety of environmental stressors, including heavy metals, which makes it an excellent model to study the effects of these pollutants on aquatic organisms (Kim *et al*. 2017). *Daphnia* typically reproduces by cyclical parthenogenesis, alternating between apomictic and sexual reproduction. Under favorable conditions, they produce direct-developing summer eggs via apomixis. However, when environmental conditions become unfavorable such as during droughts or freezing, diapausing eggs are formed through meiosis and require fertilization for development. In temporary water bodies, populations must be re-established each year from these diapausing eggs. Some *Daphnia* species reproduce by obligate parthenogenesis in which case the diapausing eggs are also produced apomictically (Hebert and Crease 1980, 1983). It is well documented that the emergence of obligate parthenogenesis in *D. pulex* resulted from hybridization between *D. pulex* and *D. pulicaria* (Hebert 1981; Xu *et al*. 2015).

The goal of this study was to evaluate how rDNA changes in mutation accumulation (MA) lines of *D. pulex* under environmentally relevant concentrations of Ni and Cu, separately and in combination. We used short-read whole-genome sequences to assess the effects of chronic Ni and Cu exposure on rDNA sequence and CNV, the rate of copy number change, and patterns of rDNA haplotype expansion in 2 MA lineages established from populations that have not been contaminated with Ni or Cu. In addition, we examined rDNA variation in 9 individuals from a metal-free, non-MA population in which natural selection was able to operate. This population originated from the same ancestral progenitor as one of the MA lineages. We also compared our results on rDNA copy number with a previous study (Harvey *et al*. 2020) that used qPCR to examine the effects of Ni and Cu on rDNA CNV in the same *D. pulex* MA lines.

## Methods

### *D. pulex* MA line genome data

To investigate rDNA variation under Ni and Cu stress, we analyzed genome sequences from a total of 127 MA lines of *D. pulex* sampled from between 75 and 204 generations of propagation (Table 1). These lines originated from 2 lineages propagated clonally from an individual collected from natural populations inhabiting ponds near Windsor, Ontario. One lineage was initiated from a cyclically parthenogenetic *D. pulex* female and is denoted as the SX (sexual) lineage (Lat. 42°18″, Long. −83°04″). The other MA lineage was initiated from an obligately parthenogenetic female that likely descended from an ancient *D. pulex–D. pulicaria* hybrid (Hebert 1981; Xu *et al*. 2015) and is denoted as the clonal hybrid (CH) lineage (Lat. 42°12″, Long. −82°98″).

**Table 1.** Genome sequences included in this study.

Lineage/group	BioProject	Treatment	Line numbers	Number of genomes	Generations of propagation
CC	PRJNA341529	Control	N/A	9	∼75
CH1	PRJNA341529	Control	001–050	28	72–89
CH2	PRJNA847774	Control	001–050	14	130–174
CH3	PRJNA847774	Control	001–050	14	161–204
CH4	PRJNA341529	Ni80	151–200	9	104–143
CH5	PRJNA341529	Cu40	251–300	9	107–123
CH6	PRJNA341529	Ni80/Cu40	351–400	9	110–121
**Total CH**				**92**	
SX1	PRJNA1156646	Control	001–050	9	104–116
SX2	PRJNA1156646	Ni80	151–200	9	94–110
SX3	PRJNA1156646	Cu40	251–300	8	95–115
SX4	PRJNA1156646	Ni80/Cu40	351–400	9	94–123
**Total SX**				**35**	
**Total**				**127**	

The CH lineage was derived from an obligately parthenogenetic *D. pulex–D. pulicaria* hybrid individual taken from a pond near Windsor, Ontario. CC is the competitive control population that was derived from the CH progenitor. The SX lineage was derived from a cyclically parthenogenetic *D. pulex* individual taken from a pond near Windsor, Ontario. Ni80 corresponds to 80 µg/uL of Ni. Cu40 corresponds to 40 µg/uL of Cu.

All mutation lines were initiated with granddaughters of the progenitor female, which were propagated apomictically and were maintained under minimum selection (Flynn *et al*. 2017). Individual females were maintained in 20 mL of FLAMES soft-water media (Celis-Salgado *et al.* 2017) and maintained at a temperature of 18°C, humidity of 70%, and light/dark cycle of 12 h:12 h. Each culture was fed a mixture of 3 algal species, *Ankistrodesmus* sp., *Scenedesmus* sp., and *Pseudokirchneriella* sp. twice each week. To initiate each generation, a single individual was haphazardly chosen from the offspring of the female that initiated the previous generation and placed in fresh medium (Flynn *et al*. 2017). In addition, a competitive control (CC) population, which experienced selection without metal exposure, was established from the CH lineage. This population was maintained in a 15-L aquarium under the same conditions of light, temperature, and food as the MA lines. After 46 months, 9 individuals were removed from this population for sequencing at which time the population size was estimated to be 100–250 individuals (Flynn *et al*. 2017).

Fifty lines of each lineage were established for each of 4 treatments: a metal-free control (lines 1–50) and 3 metal treatments consisting of 80-µg/mL Ni (Ni80, lines 151–200), 40-µg/mL Cu (Cu40, lines 251–300), and a mixture of both metals (Ni80Cu40, lines 351–400). These metal concentrations were selected because they reflect the Ni and Cu pollution levels typically found in historically contaminated lakes in the Sudbury region of Ontario, Canada, where *Daphnia* is known to inhabit (Keller and Yan 1991; Celis-Salgado *et al*. 2016). In addition, preliminary 14-day toxicity tests indicated that these concentrations do not significantly affect survival or reproduction in these lineages under the tested conditions (Cristescu *et al*. unpublished data). Therefore, they are ideal for studying the effects of chronic mild stress.

An MA line was sampled by extracting DNA from 1 to 5 daughters of the female that initiated the generation. To reduce microparasites and symbionts, the animals were treated with an antibiotic and fed Sephadex beads prior to DNA extraction (Flynn *et al*. 2017). DNA was extracted using the cetyltrimethylammonium bromide method (Doyle and Doyle 1987). Sequencing libraries were prepared with the Illumina Nextera kit and the libraries were sequenced with 100-bp paired-end reads on an Illumina HiSequation 2000 instrument by Genome Quebec at McGill University. Further details on culturing the MA experiment, tissue collection, and genome library preparation are provided by Flynn *et al*. (2017) and Bull *et al*. (2019).

We analyzed the genomes of 35 samples from the SX lineage: 9 each from the control (SX1), Ni80 (SX2), and Ni80Cu40 (SX4) treatment and 8 from the Cu40 treatment (SX3); 56 samples from the CH lineage: 56 from the control (CH1, CH2, and CH3) and 9 each from the 3 metal treatments (CH4, CH5, and CH6); and 9 samples from the CC population (Table 1; Supplementary Table 1 in Supplementary File 2). Genome sequences of the CH samples from lines exposed to metal and the control lines up to generation 89 were obtained from the NCBI Sequence Read Archive (SRA; BioProject PRJNA341529; Flynn and Bull 2016). Two additional samples from 14 previously sequenced CH control lines were obtained from BioProject PRJNA847774 (Maruki 2022). All genome sequences were obtained as FASTQ files containing the reverse and forward reads. Genome sequences from all the SX samples have been submitted to the SRA under BioProject PRJNA1156646 (Crease *et al*. 2024). Trimmomatic v 0.39 (Bolger *et al*. 2014) was used to remove adaptors. The genomic reads that had been trimmed were aligned with the reference sequences of 4 rDNA regions and 16 exons from single-copy genes using BWA mem v 0.7.17 (Li and Durbin 2009). Following this, SAMtools (Danecek *et al*. 2021) converted the resulting SAM files into sorted BAM files. Duplicate sequences were removed using Picard v 2.23.2 (Picard Toolkit 2019) with default settings. BEDTools v2.30.0 (Quinlan and Hall 2010) was then used to detect the per-base read depth of each reference sequence in the deduplicated BAM files. A summary of the read depth outputs (mean, mode, and median) was attained using stats2.sh (McElroy *et al*. 2021).

### Reference sequence for rDNA regions

The rDNA repeat was divided into 4 regions: 2 rRNA genes: 28S (4,376 bp) and 18S (2,292 bp) and 2 unique IGS regions. The first unique region (IGS1, 645 bp) was upstream of the subrepeat region (Crease 1993), while the second unique region (IGS2, 3,170 bp) was downstream of the subrepeat region. Due to low read depth at the ends of the IGS sequences, IGS1 was trimmed to 571 nt and IGS2 was trimmed to 3,123 nt. The IGS subrepeats were excluded as it is not possible to differentiate them from each other using short-read sequence data.

Sequences were obtained from GenBank and included the *D. pulex* 18S rRNA gene (accession AF014011; Crease and Colbourne 1998), the *D. pulicaria* 28S rRNA gene (accession AF346514; Omilian and Taylor 2001), and a *D. pulex* IGS sequence (accession L07948.1; Crease 1993). Sequences for 16 exons from single-copy genes that were used to estimate rDNA copy number were obtained from the *D. pulex* genes (V1.0) dataset in Ensembl BioMart (https://metazoa.ensembl.org/Daphnia_pulex_gca021134715v1rs/Info/Index). Details on the choice of these exons are described in Elguweidi and Crease (2024). All reference sequences are available in Supplementary File 1.

### Estimating the haploid copy number of rDNA

Genome sequences of all genomes were mapped to the 4 rDNA and 16 exon reference sequences as described above. The haploid copy number of each rDNA region was estimated by dividing the mean read depth of the rDNA region by the mean read depth of the 16 exons. To estimate the rate of copy number change per generation in the 28S gene, hereafter shortened to change rate, we divided the difference in diploid copy number between generation 0 and the sampled generation by the sampled generation. We used the median copy number of 28S in the control samples to represent the lineage progenitors at generation 0. Additionally, 14 CH control lines were sampled at 3 time points (Table 1): approximately generation 82 (CH1), 159 (CH2) and 188 (CH3), which allowed us to estimate the rate of copy number change over 3 intervals: ∼77 generations between sample 1 and sample 2; ∼106 generations between sample 1 and sample 3; and ∼30 generations between sample 2 and sample 3. Rates based on generation 0 were not included in this analysis.

### Metal effects

We examined the distribution of copy numbers and change rates within each treatment group using the Shapiro–Wilk test. If the Shapiro test indicated that the copy number or change rates across treatments within each lineage did not follow a normal distribution, we applied the nonparametric Kruskal–Wallis test to investigate the significance of differences between the median values. If the Shapiro test was not significant, we performed an analysis of variance (ANOVA).

### Variant calling

We used BCFtools (version 1.11) to identify single nucleotide polymorphisms (SNPs) within the rDNA regions, generating an mpileup file for each genome. Subsequently, R (R Core Team 2020) was employed to process the mpileup files, producing read depth summaries for each of the 4 nucleotides. Allele counts <5 at a SNP site within a sample were set to 0, and a recalculated total read depth was determined for that site. SNPs within a sample with a total read depth < 99 were disregarded. The allele frequency of each SNP within each sample was calculated by dividing the count of reads for each nucleotide by the total read depth, which includes all 4 nucleotides. A SNP was excluded from further analysis if the mean frequency of allele 1 (the reference allele) was more than 0.990 across all samples in a lineage. We used the secondary structure of the *D. pulex* 18S gene to determine the location of SNPs in this gene (Crease and Colbourne 1998). We localized SNPs in the 28S gene by aligning the *D. pulicaria* sequence with the *Drosophila melanogaster* sequence (accession number M21017.1). The location of helices in the *D. melanogaster* sequence, as identified by Wuyts *et al*. (2001), was used for this alignment.

### Haplotype estimation

We aimed to detect haplotypes and determine their frequency within each sample by analyzing SNP allele frequencies in each lineage independently using the R package, haploSep (Pelizzola *et al*. 2021). Before running haploSep, we used haploSelect to identify the anticipated number of haplotypes in each sample. However, if this number exceeded the log2 of the number of SNPs, we used the smaller number in haploSep as suggested by Pelizzola *et al*. (2021). In addition, the “bias” parameter was set to FALSE so that haplotype frequencies within individuals would sum to 1, which means that minor haplotypes that include low-frequency variants may not have been identified. However, using these 2 settings gave identical results in multiple runs of the program.

We generated a haplotype network based on the number of nucleotide differences at the 59 SNPs across both MA lineages using the Templeton, Crandall, and Sing (TCS) method (Templeton *et al*. 1992) and the program, PopArt (https://popart.maths.otago.ac.nz/).

We calculated the expected heterozygosity (H_e_) within samples based on the haplotype frequencies generated by haploSep using the equation, 1 − (Σ*p_i_*^2^) where *p* is the frequency of the *i*th haplotype in a sample. We then performed a linear regression between H_e_ and the diploid number of 28S genes in R.

## Results

### Cyclic parthenogen (SX) lineage

#### Diploid copy number of rDNA

**Fig. 1.**
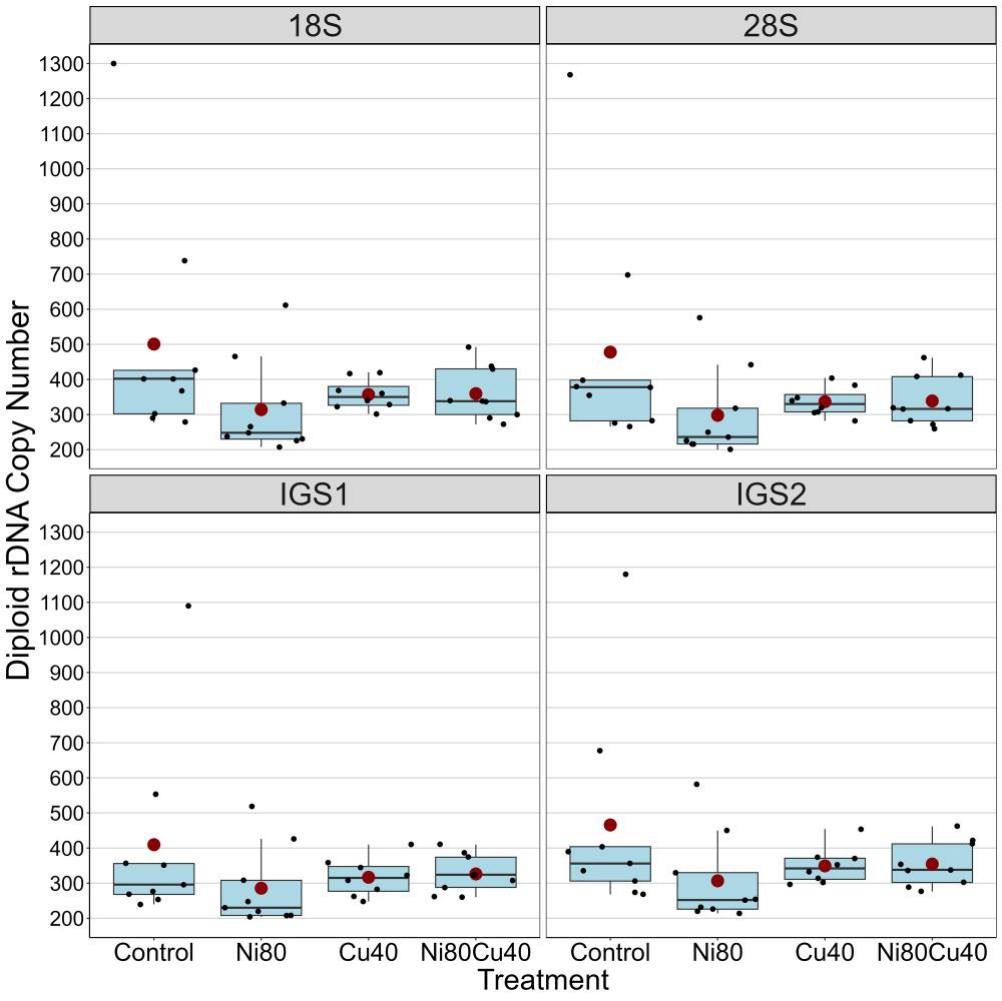
Distribution of diploid copy number across rDNA regions (18S, 28S, IGS1, and IGS2) under 4 treatments in 35 samples of the SX MA lineage. SX is the cyclically parthenogenetic (sexual) *D. pulex* lineage. Each box represents the IQR, the line inside the box indicates the median, the whiskers extend to 1.5 times the IQR, and individual data points are shown as dots. Large red circles denote the mean values for each rDNA region. Outliers beyond the whiskers are also indicated.

The diploid copy number of the 18S gene in the 35 samples from the cyclic SX lineage varied from 208 to 1,300 (mean = 383.5; Supplementary Table 2 in Supplementary File 2). The 28S gene varied from 200 to 1,268 copies (mean = 363.4), IGS1 varied from 204 to 1,068 copies (mean = 335.0), and IGS2 varied from 214 to 1,180 copies (mean = 369.6) (Fig. 1; Supplementary Table 2 in Supplementary File 2). Three samples, SX016_116, SX020_115, and SX173_110 (the number after the dash indicates the generation at which the line was sampled), showed notably higher rDNA copy numbers compared to the remaining samples. These samples were considered outliers as they fall outside the range of 1.5 * interquartile range (IQR) (Fig. 1). The diploid copy numbers of the 28S and 18S genes were strongly correlated (*P* < 0.001) with an *R*^2 =^ 0.999 (Supplementary Fig. 1 in Supplementary File 3) and a regression coefficient slightly lower than the expected value of 1 (0.972). IGS1 and IGS2 copy numbers were also highly correlated (*R*^2^ = 0.991, *P* < 0.001) with a regression coefficient = 0.888 (Supplementary Fig. 1 in Supplementary File 3).

We estimated the diploid 28S copy number of the SX progenitor (378) as the median value for the control samples. The change rate from generation 0 to the sampled generation ranged from −1.68 to 7.74 copies with a mean of −0.17, which is not significantly different from 0 (*t*-test = 0.61, *P* = 0.55). This suggests that there is no significant bias in the direction of copy number change (Supplementary Fig. 2 in Supplementary File 3). The absolute change rate ranged from 0 to 7.74 with a mean of 1.03 copies per generation (Supplementary Fig. 2 in Supplementary File 3).

#### Metal effects

The Shapiro–Wilk test for normality of diploid 28S copy numbers yielded a significant result (*P* < 0.001) so we performed the nonparametric Kruskal–Wallis test. The test indicated no significant differences in median 28S copy number among the treatments with *P*-values of 0.147 for the full dataset and 0.203 when the 3 outlier samples were excluded from the analysis (Fig. 1; Supplementary Table 3 in Supplementary File 2).

Despite the lack of significant differences in median 28S copy number among treatments, the range of absolute change rates was greater in the control and Ni80 treatments than the other 2 treatments. Furthermore, the median absolute change rate was higher in the Ni80 and Ni80Cu40 treatments compared to Cu40. To determine if there was a significant treatment effect on the absolute change rate, we square root transformed the data. The transformed data were not normally distributed (*P* = 0.002), but there was 1 outlier (SX020_115) identified based on the *Q*–*Q* residuals. After excluding this sample, the Shapiro–Wilk test was not significant (*P* = 0.974), so we performed an ANOVA, which yielded significant results (*F*-stat = 3.87, *df* = 3, 30, *P* = 0.019). Subsequently, we conducted a Tukey test, which showed that the mean absolute change rate in the Ni80 treatment was significantly higher than the control (Supplementary Table 3 in Supplementary File 2; *P* = 0.046) and the Cu40 treatment (*P* = 0.023).

#### rDNA variation

**Fig. 2.**
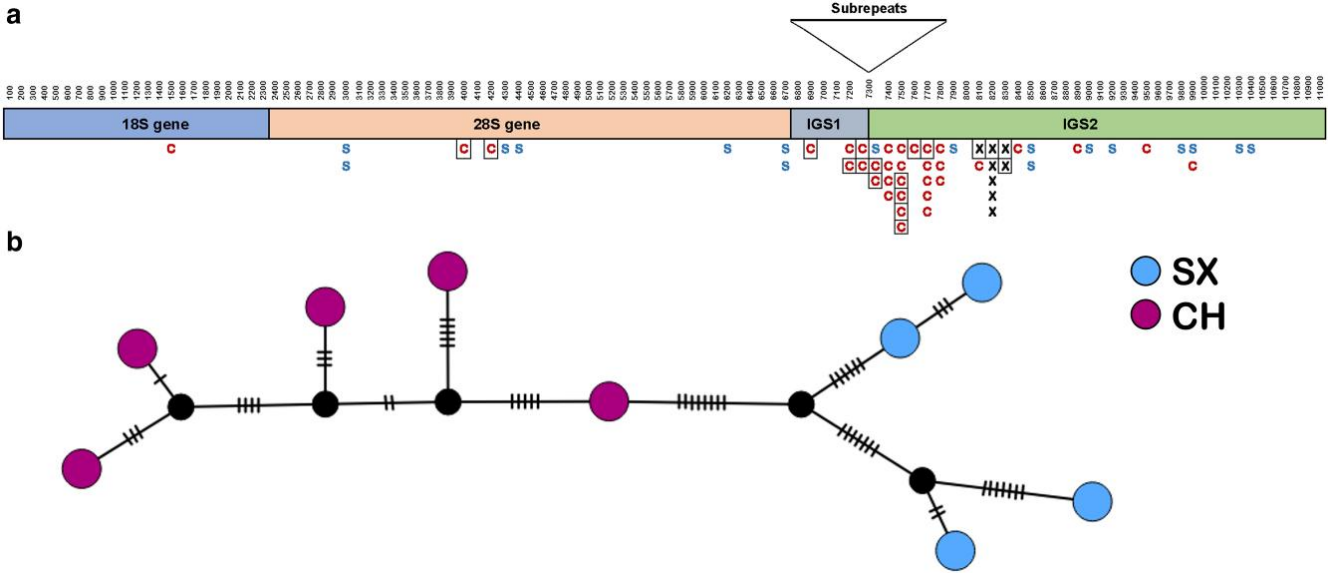
SNP and haplotype variation in the rDNA repeat of 127 samples from 2 lineages of *D. pulex* MA lines. SX (35 samples) is the sexual *D. pulex* lineage. CH (83 samples) is the clonal, hybrid (*D. pulex* × *D. pulicaria*) lineage. CC (9 samples) is the CC population established from the same individual as the CH lineage. a) Distribution of 59 SNPs across the rDNA repeat. The rDNA repeat unit is divided into 100-nt windows. The subrepeat region between the IGSs (IGS1 and IGS2) was omitted from analysis. This region is composed of a variable number of 3 subrepeat types: subrepeat A (184–222 nt), subrepeat B (96–100 nt), and subrepeat C (184–194 nt) (Ambrose and Crease 2011). Black Xs (8) are SNPs identified in both lineages. Blue Ss (17) are SNPs identified only in SX samples. Red Cs (34) are SNPs only identified in CH or CC samples. Allele 2 was the most common (or only) allele at 2 sites in the 28S gene, 4 sites in IGS1, and 8 sites in IGS2 (boxed) in the CH lineage. b) TCS haplotype network based on the 59 SNPs. Each circle represents a unique haplotype. The number of hash marks indicates the number of nucleotide differences between haplotypes.

Twenty-five SNPs identified in the SX samples met our retention criteria. These SNPs were found only in IGS2 and the 28S gene. IGS2 had 18 SNPs, 8 of which were shared with the CH lineage. The 28S gene had 7 SNPs, located in the variable regions: 2 in V2, 2 in V6, 2 in V12, and 1 in helix G18 (Fig. 2a).

**Fig. 3.**
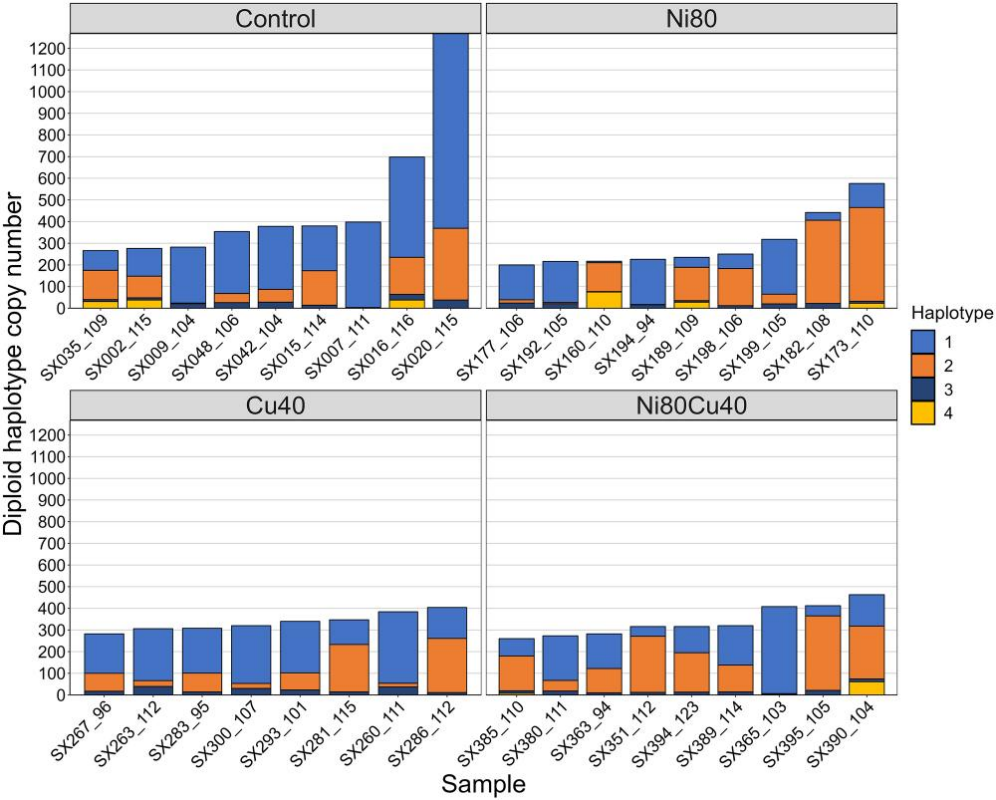
Distribution of 4 haplotypes in 35 samples of the SX MA lineage from 4 treatments. Lines 0–50 are controls (SX1 in Table 1), lines 150–200 were exposed to Ni (SX2), lines 251–300 were exposed to Cu (SX3), and lines 351–400 were exposed to both Ni and Cu (SX4). The number after the underscore is the generation at which the line was sampled. The samples are ordered by the total diploid 28S copy number.

HaploSep identified 4 haplotypes based on the 25 SNPs (Supplementary File 4). The number of differences between haplotypes in the SX lineage ranged from 3 to 23 with a mean of 14.7 (Fig. 2b). Haplotype 1 was most common among the control samples and Cu40 treatment, while haplotype 2 was most prevalent in the Ni80 treatment. When the total 28S copy number in a line increased, not all haplotypes increased. For example, haplotype 1 tended to increase more than the others (Fig. 3). A regression analysis of the expected heterozygosity (H_e_) within samples and their diploid number of 28S genes was not significant (*R*² = −0.030, *P* = 0.934); samples with high copy number exhibited a level of variation comparable to that of samples with low copy number (Supplementary Fig. 3 in Supplementary File 3).

### Obligate parthenogen (CH) lineage

#### Diploid copy number of rDNA

We noticed a substantially lower copy number for IGS1 compared to the other rDNA regions, leading us to examine the read depth across the sequence. We identified regions within IGS1 that failed to map to the reference sequence, causing a decrease in average read depth (Supplementary Fig. 4 in Supplementary File 3). Furthermore, we discovered that ∼300 nt at the 5′ end of IGS2 had low or 0 read depth. However, we did not observe 0 read depth in these regions in the SX samples (Supplementary Fig. 4 in Supplementary File 3). Consequently, we decided to exclude IGS1 from the rDNA copy number estimations and removed the 300 nt from IGS2 when estimating copy number in the CH samples. Further details about the CH dead zone in IGS1 are provided in Supplementary Fig. 5 in Supplementary File 3.

**Fig. 4.**
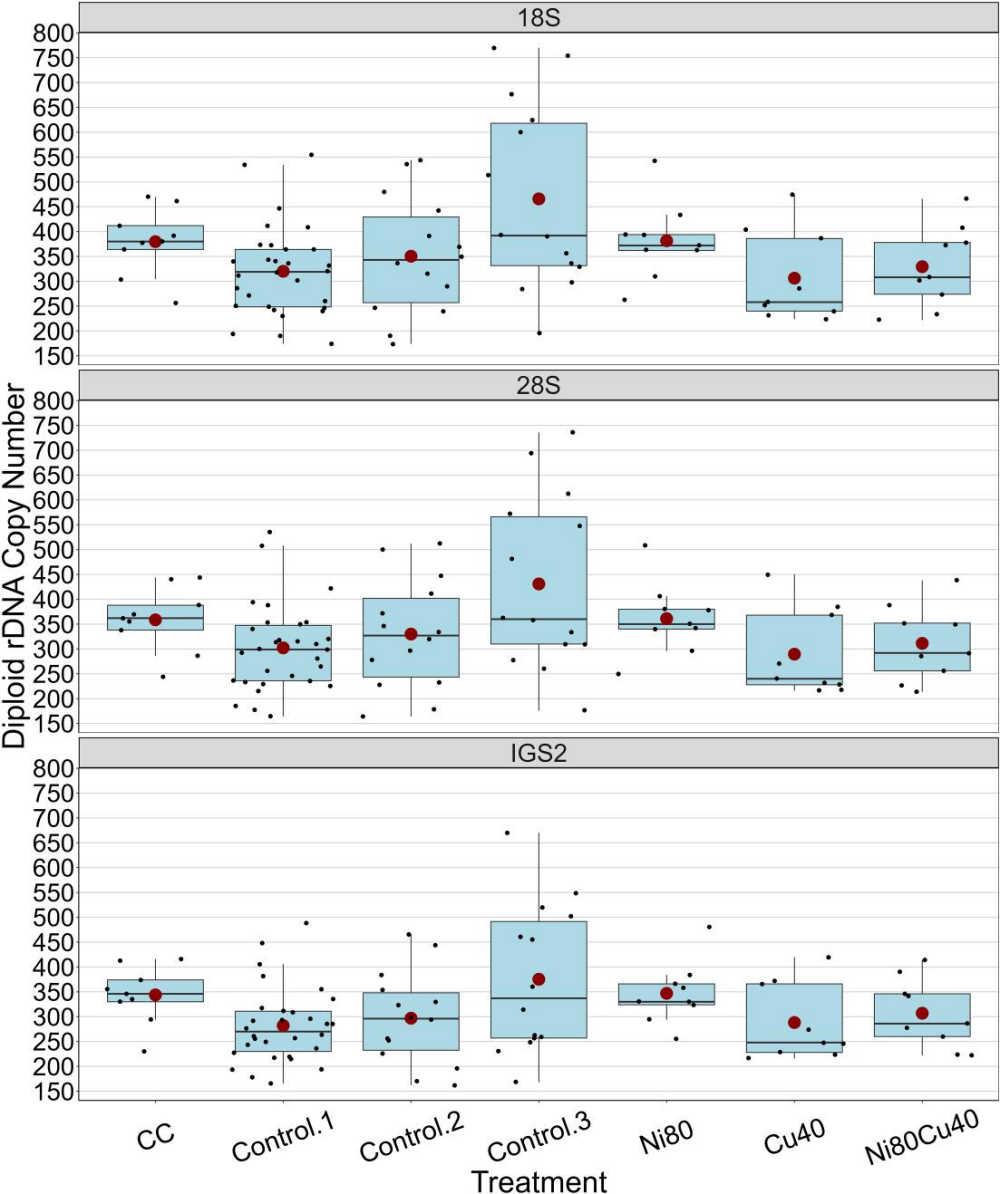
Distribution of diploid copy number across rDNA regions (18S, 28S, and IGS2) and 4 treatments in 92 samples of the CH MA lineage. Each box represents the IQR and the line inside the box indicates the median. The whiskers extend to 1.5 times the IQR, and individual data points are shown as dots. Large red circles denote the mean values for each rDNA region. Outliers beyond the whiskers are also indicated.

The diploid copy number in the CC samples ranged from 256 to 470 for 18S (mean = 189.9; Supplementary Table 2 in Supplementary File 2), from 244 to 444 for 28S (mean = 179.3), and from 250 to 450 for IGS2 (mean = 186.7) (Fig. 4, Supplementary Table 2 in Supplementary File 2). The diploid rDNA copy number for 18S in the CH samples (Fig. 4) varied from 174 to 770 copies (mean = 358.5), from 164 to 736 copies for 28S (mean = 337.2), and from 174 to 714 for IGS2 (mean = 339.6). The third set of control samples (around generation 188) had a higher mean and a wider range than the other control groups (around generations 83 and 159). Therefore, we performed an ANOVA test on the diploid 28S copy number of these 3 groups, and the results were significant (*F*-stat = 4.527, *df* = 2,53, *P* = 0.015). Subsequently, we applied a Tukey test, which revealed a significant difference between the means in the first and third control groups (*P* = 0.010).

A regression analysis revealed a significant relationship between the diploid copy number of 28S and 18S genes (Supplementary Fig. 6 in Supplementary File 3), with a *P* < 0.0001, an *R*² = 0.997, and a regression line slope slightly lower than the expected value of 1 (0.930). The regression analysis indicated that the relationship between IGS2 and 18S was also significant (*R*² = 0.950, *P* < 0.0001; Supplementary Fig. 6 in Supplementary File 3).

We estimated the diploid 28S copy number of the CH progenitor using the median value from the first control group (∼83 generations, median = 299). We then calculated the change rate from generation 0 to the sampled generation in the CC samples (approximately generation 75), which ranged from −0.73 to 1.93 with a mean of 0.79 (Supplementary Fig. 7a in Supplementary File 3), which was significantly different than 0 (*t*-test = 2.753, *P* = 0.024). The absolute values ranged from 0.17 to 1.93 with a mean of 0.99 (Supplementary Fig. 7b in Supplementary File 3). Additionally, the change rate for CH samples from generation 0 to the sampled generation ranged from −1.68 to 3.11 with mean of 0.22 (*t*-test = 0.699, *P* = 0.486; Supplementary Fig. 7a in Supplementary File 3), which was not significant. The absolute value ranged from 0.012 to 3.11 with a mean 0.716 (Supplementary Fig. 7b in Supplementary File 3).

The change rate was also estimated for all combinations of samples within the 14 control lines (CH1, CH2, and CH3 in Table 1) that were sampled at 3 time points (Supplementary Table 4 in Supplementary File 2). The values ranged from −7.54 to 14.67 with a mean of 1.60, which is significantly higher than 0 (*t*-test = 2.06, *P* = 0.045). We also estimated the mean separately for the 3 intervals. The mean change rate (3.72) for the shortest interval (∼30 generations between the second and third samples) was not significantly different from 0 (*t*-test = 1.74, *P* = 0.097) nor was the mean change rate (0.04) for the intermediate interval (∼77 generations between the first and second sample) (*t*-test = 0.06, *P* = 0.953). However, the mean change rate for the longest interval (∼106 generations between the first and last sample) was 1.04, which was almost significant (*t*-test = 2.07, *P* = 0.058). When considering only the direction of change between samples in the 14 lines (excluding generation 0), there were 17 increases and 11 decreases (*χ*^2^ = 1.286, *P* = 0.257, *df* = 27). Even though all 3 means are positive and there are more increases than decreases, the only statistically significant bias toward increased copy number was observed across all 42 values from the 14 control lines. This was likely driven by the larger mean copy number at generation 188 compared to generation 83.

**Fig. 5.**
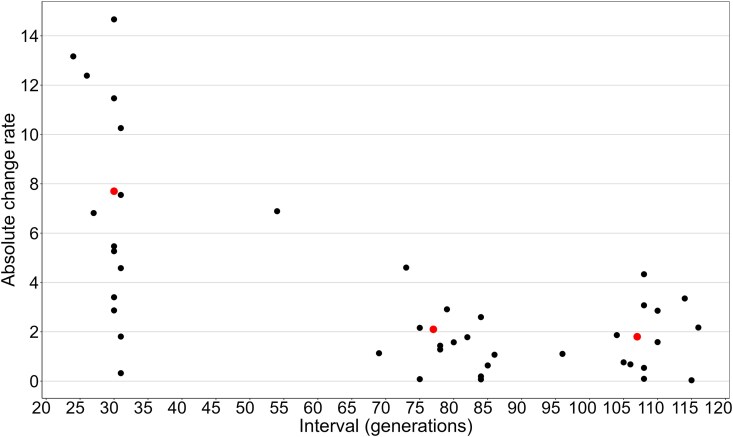
The absolute diploid 28S copy number change rate per generation across 14 CH control lines that were sampled at 3 time points (CH1, CH2, and CH3 in Table 1). The absolute change rates were calculated based on differences in copy number between these 3 time points. Each black dot represents an individual measurement. The red dots indicate the mean absolute change rate for 3 interval groups (∼30 generations, ∼77 generations, and ∼106 generations).

Absolute change rate between samples from each of the 14 control lines sampled 3 times (CH1, CH2, and CH3) varied from 0.32 to 14.67 (Fig. 5). Moreover, the range of values at the shortest time interval (∼30 generations) was substantially larger than the range at longer intervals, as was the mean (7.7 for 30 generations, 2.0 for 77 generations and 1.8 for 106 generations). The *t*-test comparing mean absolute change rate between the short and intermediate intervals was significant (*t*-test = 5.61, *P* < 0.001).

#### Metal effects

**Fig. 6.**
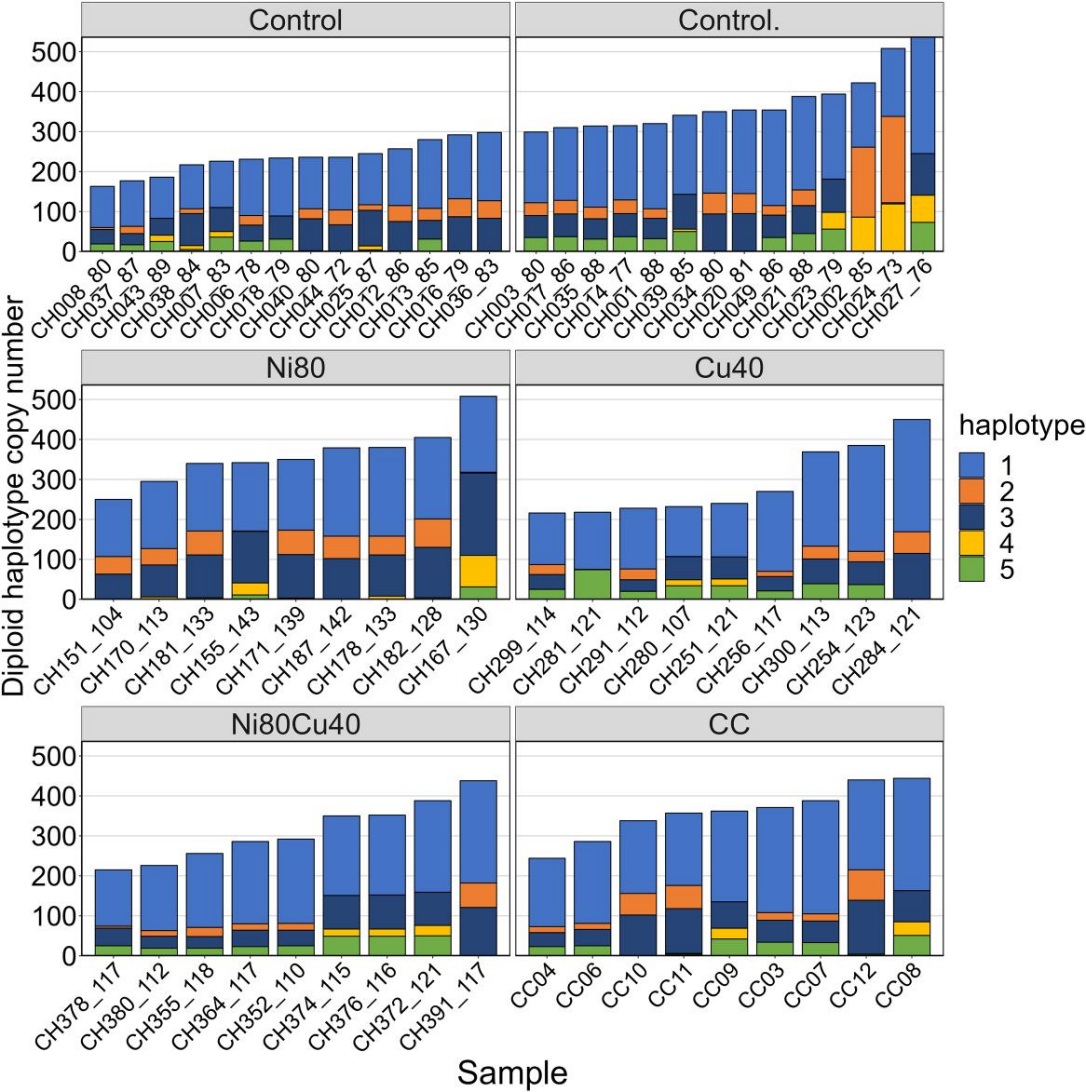
Distribution of 5 haplotypes in 64 samples of the CH MA lineage. Lines 0–50 are controls (CH1, CH2, and CH3 in Table 1), lines 150–200 were exposed to Ni (CH4), lines 251–300 were exposed to Cu (CH5), and lines 351–400 were exposed to both Ni and Cu (CH6). The number after the underscore is the generation at which the line was sampled. The samples are ordered by the total diploid 28S copy number. Only the 28 control lines sampled at ∼generation 83 are included in this plot.

The Shapiro–Wilk test was significant, indicating that the diploid 28S copy numbers did not follow a normal distribution. Thus, we used the nonparametric Kruskal–Wallis test, which was not significant (*P* = 0.342), suggesting that there were no significant differences between the median 28S copy number of treatment groups (Fig. 6; Supplementary Table 3 in Supplementary File 2). However, when we replaced the 28 control samples from approximately generation 83 (CH1) with the 14 control lines sampled at approximately generation 159 (CH2), the Shapiro–Wilk test was not significant (*P* = 0.109). Consequently, we performed an ANOVA, but it was also not significant (*F*-stat = 1.401, *df* = 3, *P* = 0.258) suggesting that there were no significant differences in mean 28S copy number between the control group sampled at approximately generation 159 (CH2) and the treatment groups (CH4, CH5, and CH6).

The 28 CH control lines (CH1) exhibited a wider range of absolute change rates than the metal treatment lines (CH4, CH5, and CH6; Supplementary Fig. 7 in Supplementary File 3). A Shapiro–Wilk test of the square root transformed rates for the metal lines and the 28 control lines sampled at approximately generation 83 was not significant (*P* = 0.727). Consequently, we performed an ANOVA to test for significant differences among treatments, which also yielded no significant results (*P* = 0.494). Since the 28 control samples were taken earlier than the metal-exposed samples (approximately generation 121), we substituted the 28 controls sampled at approximately generation 83 with the 14 control lines sampled at approximately generation 159 (CH2), including the outliers CH008_153 and CH012_155. A Shapiro–Wilk test on these new control samples was significant (*P* < 0.001) so we conducted a Kruskal–Wallis test, which was not significant (*P* = 0.355). However, after removing the outliers, the Shapiro–Wilk test was not significant (*P* = 0.968), so we performed an ANOVA, which was also not significant (*P* = 0.26). This indicates no significant difference in the mean absolute change rate among treatments.

#### rDNA variation

We detected 5 haplotypes based on a total of 42 SNPs (Supplementary File 4). Among these, 34 were unique to the CH lineage, including 5 SNPs in IGS1, one in the 18S gene and two in the 28S gene. The remaining unique SNPs were located in IGS2 (Fig. 2a). The 18S SNP was located in helix 37. The 28S SNPs were located in variable regions V5 and V6. Allele 2 was fixed or nearly fixed in 14 of the unique SNPs. The number of nt differences between haplotypes in the CH lineage ranged from 4 to 14 with a mean of 10.8 (Fig. 2b). Although 8 SNPs were shared with the SX lineage (Fig. 2a), the haplotypes from each lineage formed distinct clusters (Fig. 2b).

The haplotypes differed substantially in their distribution between samples (Fig. 6). Haplotype 1 dominated across all treatments and the controls sampled at approximately generation 83 (CH1), although its level of dominance varied within treatments. Haplotype 2 was also common but did not reach the same level of dominance as haplotype 1. Haplotypes 3–5 generally occurred at lower frequencies, with haplotype 3 displaying more variability in frequency than haplotypes 4 and 5 (Fig. 6). When the total 28S copy number increased, not all haplotypes increased proportionally. The regression analysis between the diploid 28S copy number and H_e_ was significant (*R*² = 0.07, *P* = 0.006), with a positive slope of 0.00014 (Supplementary Fig. 8 in Supplementary File 3), suggesting that lower frequency haplotypes tended to expand more than the common haplotype when copy number increased.

**Fig. 7.**
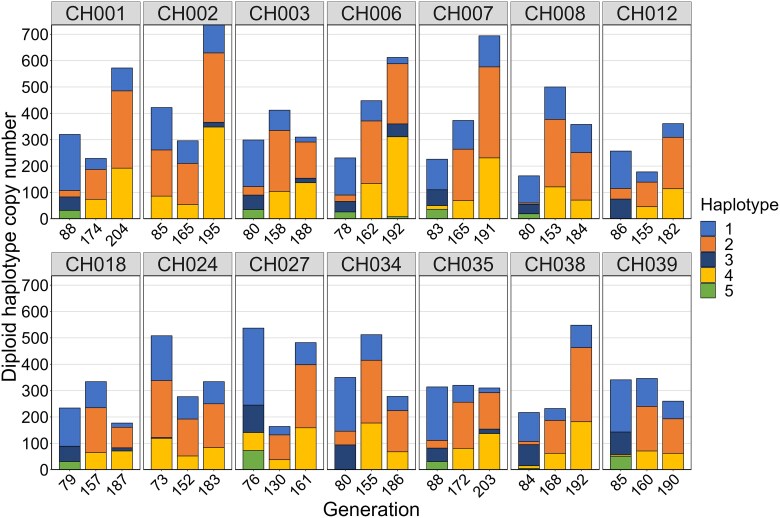
Distribution of 5 haplotypes in 42 samples of 14 CH control lines sampled at 3 generations. Haplotype number is based on the diploid 28S copy number.

A notable shift in haplotype dominance was apparent from generation 83 to later generations in the 14 control lines sampled 3 times (Fig. 7). For instance, haplotype 3, which was prevalent at generation 83, was much less dominant by generations 159 and 188. Conversely, haplotypes 2 and 4 became very common in generations 159 and 188.

## Discussion

### rDNA CNV

We observed variation in rRNA gene numbers in MA lines of *D. pulex* (SX, CH) propagated by apomictic reproduction and a CC population (maintained under selection) belonging to the CH lineage (CC). Some SX samples were extreme outliers (e.g. SX016_115, SX020_116, and SX173_110) with diploid copy numbers reaching up to 1,300. In contrast, the highest diploid copy number in the CH lineage was 770 (CH002_195). The mean rDNA copy number in the CC samples was higher than that in both SX and CH and exhibited lower variance compared to both. Regression between 18S and 28S copy numbers in both the SX and CH lineages showed strong correlations, as did regression between IGS1 and IGS2 in SX.

Comparing rDNA copy numbers between cyclically parthenogenetic *D. pulex* populations in ponds from the MidWest USA and MA lines reveals that some natural populations, such as PA and BUS both of which are cyclic parthenogens, exhibit a broad range of rDNA copy numbers, with higher averages (Elguweidi and Crease 2024). In contrast, the MA lines generally displayed lower rDNA copy numbers with less variation between individuals. However, the presence of some MA samples with rDNA copy numbers as high as those in natural populations suggests that significant genetic variation can arise during a relatively short period of time (∼80 generations).

The variability in rDNA copy number in our study is comparable to that observed by Harvey *et al*. (2020), who investigated rDNA CNV in the same MA lines using qPCR. Although all samples in Harvey *et al*. were taken at different generations than ours, the overall variability is comparable (Supplementary Table 5 in Supplementary File 2). Harvey *et al*. (2020) found the lowest diploid 18S copy number in the CH lineage to be 76 copies, whereas the lowest was 174 in our study. Similarly, Harvey *et al*. (2020) reported a maximum 18S diploid copy number of 867, whereas the maximum in our study was 770. In the SX lineage, Harvey *et al*. (2020) noted a minimum diploid copy number of 108, compared to our minimum of 208. Their maximum was 771, whereas ours was 1300, which is notably higher and was considered an extreme outlier. However, the next highest value in our study, 738, closely aligns with the maximum reported in the SX lineage by Harvey *et al*. (2020).

The similarity in the range of results based on Harvey *et al*.'s data using qPCR and our data using next-generation sequencing (NGS) suggests that both methods capture substantial genetic variation. However, NGS may be better able to detect extremely high values because qPCR may reach a saturation point, rendering it unreliable for quantifying very large copy numbers (Morton *et al*. 2023). In addition, qPCR has limitations that can affect the accuracy of copy number estimates, including primer biases that can lead to overestimating or underestimating copy number. Moreover, the qPCR reactions with each primer pair are set up individually, providing the opportunity for variation in copy number estimates due to slight differences in the amount of template added to each reaction. In addition, the qPCR estimates were based on only 2 single-copy genes. Despite these limitations, Harvey *et al*. (2020) found a strong correlation (*R*^2^ = 0.72, *P* = 0.001) between the duplicate 18S copy number estimates in 19 samples.

Other studies have examined changes in rDNA copy number in MA lines. For instance, Averbeck and Eickbush (2005) measured rDNA copy number on the X chromosome in 16 MA lines of *D. melanogaster* after 400 generations and found it to vary from 140 to 310. Assuming this represents the copy number per X chromosome (i.e. haploid), this range is similar to the variation we observed in the *D. pulex* MA lines; however, the variation in *Daphnia* was generated in <200 generations. Averbeck and Eickbush (2005) suggested that significant variation in *Drosophila* rDNA locus size, similar to that seen in natural populations, can occur rapidly even in a controlled laboratory setting. Similarly, Bik *et al*. (2013) estimated the haploid rDNA copy number in 4 MA lines of *Caenorhabditis elegans* and reported an increase compared to the parent N2 strain. While the parent strain had about 112 (224 diploid) rDNA repeats, the MA lines showed an increase from 120 to 256 (240–512 diploid) repeats over 250–420 generations.

The mean rate of change in 28S copy number per generation from generation 0 was not significantly different from 0 in both the CH (0.22) and SX (−0.17) lineages. We also found that the number of increases and decreases (17 and 11) between time points in the 14 CH lines sampled 3 times was not significantly different from the expected 1:1 ratio. We applied the same approach to estimate change rate of diploid 18S copy numbers in CH lines sampled more than once by Harvey *et al*. (2020) and found that the mean rate was −2.14, which is significantly lower than 0 (*t*-test = −4.455, *P* = 0.001) (Supplementary Fig. 9 in Supplementary File 3). Moreover, there were significantly more decreases than increases (23 increases, 54 decreases, *χ*^2^ = 12.48, *P* = 0.0004), which was due to more decreases in lines from the metal treatments (the difference was not significant in the controls). The mean change rate in SX lines sampled more than once by Harvey *et al*. (2020) was −0.83 with 19 increases and 20 decreases (*χ*^2^ = 0.026, *P* = 0.87).

Overall, the results from both studies suggest that changes in *D. pulex* rDNA copy number are not strongly biased in direction, at least in the absence of metal exposure (see below). In contrast, Bik *et al*. (2013) found that rDNA copy number increased in all MA lines of *C. elegans* after 250–420 generations. Although diploid copy numbers as low as 70 have been reported in wild isolates of *C. elegans* (Morton *et al*. 2023), lower copy numbers in lab strains were linked to developmental abnormalities and reduced fertility (Morton *et al*. 2023). Therefore, the observed increase in copy number in the *C. elegans* MA lines was likely not due to viability issues, as the parent strains had high enough initial copy numbers that any reductions would still have allowed normal viability. However, this bias toward increased copy number could also be influenced by sample size, as assaying a small number of MA lines could lead to an overrepresentation of copy number increases. Alternatively, there may be an actual bias in the direction of copy number change in this species (Bik *et al*. 2013).

The 14 CH control lines that were sampled at 3 time points (CH1, CH2, and CH3) showed that the mean and variance of the absolute change rate was higher in the shorter (30 generations) than the longer intervals (77 generations and 107 generations). This was also the case with lines that were sampled more than once by Harvey *et al*. (2020); the mean and variance of absolute change rate in CH and SX lines decreased with increasing interval length (Supplementary Fig. 9 in Supplementary File 3). This suggests that rDNA copy number changes more rapidly over shorter timescales and stabilizes over longer periods. This is consistent with the results of Bik *et al*. (2013), who estimated the rDNA change rate in *C. elegans* MA lines to be 0.03–0.34 copies per generation over 250–420 generations, which is lower than our results in *Daphnia* after 80 generations or more (mean absolute change rate was 1.03 copies per generation in the SX lineage and 0.716 in the CH lineage). Moreover, Rabanal *et al*. (2017) found that rDNA copy number in MA lines of *A. thaliana* exhibited high instability, with changes accumulating within as few as 30 generations. These findings suggest that rDNA copy number can fluctuate rapidly over short timescales, although the full extent of these fluctuations may be masked when the change rate is measured over a longer period.

Based on the results from the *Daphnia* MA lines, rates of copy number change over long intervals are substantial underestimates, as they do not reflect how much change can occur in a short period of time. While some lines have not yet experienced recombination events that change rDNA copy number, others have experienced very large changes. Indeed, significant changes in rDNA copy number within a few generations have been reported in other organisms. In *A. thaliana*, the disruption of CAF1 function led to a progressive loss of 45S rDNA, with only 10–15% of the original number of repeats remaining by the fifth generation of mutants (Mozgová *et al*. 2010). In *Saccharomyces cerevisiae*, the rapid response and dynamic regulation of rDNA copy number through the formation and reinsertion of extrachromosomal rDNA circles illustrate how organisms can quickly adapt to and correct rDNA copy number changes (Mansisidor *et al*. 2018). On the other hand, it appears that some of the large changes that occur over short intervals can be offset by subsequent events in the opposite direction, so the longer the interval, the lower the change rate for any given difference in copy number between 2 time points. Thus, change rates will tend to stabilize over long intervals if rates of recombination remain relatively constant and there is little bias in the direction of change. Thus, measuring change rate over short intervals is necessary if the goal is to determine whether rDNA copy number changes result from a few major recombination events or many small ones. Indeed, Ganley and Kobayashi (2011) found that rDNA copy number change in *S. cerevisiae* can occur as often as once per cell division and large deletions are not uncommon. Our results suggest that major changes also occur in *Daphnia* rDNA, but confirming this will require assessing MA lines at even shorter intervals.

### Impact of metal exposure

Heavy metal contamination is a threat to the health and survival of aquatic organisms due to its persistence in the environment and its ability to bioaccumulate (Kim *et al*. 2017). The toxicity of heavy metals such as Cu and Ni has been linked to the disruption of oxidative phosphorylation, depletion of glutathione, inhibition of antioxidant enzymes, production of ROS, DNA damage and the inhibition of repair mechanisms, and protein misfolding disorders (Chiarelli and Roccheri 2014). Chronic exposure to metals can also disturb the cell's own regulation of rDNA copy number. This regulatory system involves inhibiting rDNA replication during the S phase of the cell cycle, which can lead to unequal recombination events resulting in copy number changes (Kobayashi *et al*. 2004). In addition, DNA strand breaks and genomic stress could cause rDNA copy number loss as has been observed in species such as *B. nigra*. Waters and Schaal (1996) found that *B*. *nigra* exposed to a 2-h heat shock at 40°C lost many rDNA copies after just 1 generation. The authors suggested that the loss of rDNA repeats after heat shock could be due to a repair pathway similar to the recision-reannealing mechanism described by Ozenberger and Roeder (1991) for *S. cerevisiae*. In this pathway, double-stranded breaks in rDNA are repaired by creating single-stranded DNA tails, which reanneal and result in repeat loss due to the shortening of the DNA sequence.

We did not detect significant changes in mean rDNA copy number in the *Daphnia* MA lines exposed to Ni and Cu compared to the controls. Mean copy number in samples from these 2 treatments was about 30% lower than that in control samples at the same generation (Supplementary Table 6 in Supplementary File 2). In contrast, mean copy number was higher in samples from the Ni80 treatment than in the controls in this study and at generation 70 in Harvey *et al*. (2020). Copy number was also higher in samples from the Ni80Cu40 treatment in Harvey *et al*. (2020) although the differences were not significant. We observed a similar pattern of lower copy number in samples from the metal treatments compared to controls in the SX lineage, although none of the differences were significant in either study.

Although there was no significant difference in the metal treatment means compared to the controls, we did find that the mean absolute change rate was significantly higher in SX lines from the Ni80 treatment, which also had the lowest mean copy number. This finding suggests that Ni may have affected the rate of recombination and/or the length of misalignment between recombining partners during unequal crossing-over events. On the other hand, even if the recombination rate changed due to the presence of Ni, the overall direction of copy number change did not appear to shift significantly such that mean copy number was not affected. To confirm that Ni does influence recombination rate, it would be necessary to assess rDNA copy number change in MA lines at much shorter intervals.

Chain, Flynn, *et al*. (2019), and Bull *et al*. (2019) used the same *D. pulex* CH MA lines to examine rates of mutation under metal exposure in other regions of the genome. Chain, Flynn, *et al*. (2019) focused on rates of mutation involving large-scale deletions and duplications that cause CNV. They reported that the Ni80Cu40 treatment resulted in triple the rate of mutation causing CNVs that do not overlap genes and quadruple the rate of mutation causing CNVs that overlap gene regions (gene CNVs) compared to controls. Conversely, Ni and Cu alone had a moderate to no measurable effect. Chain, Flynn, *et al*. (2019) hypothesized that the elevated rate of CNVs observed under exposure to a mixture of Ni and Cu is primarily driven by an increase in double-strand breaks (DSBs) in the germline, caused by ROS generated by metal-induced stress. These DSBs increase opportunities for recombination and errors in DNA repair, particularly through nonhomologous end joining, an error-prone repair mechanism that metals may interfere with and thus cause more CNVs. Although homologous recombination is generally more accurate, it can also produce errors under stress. Chain, Flynn, *et al*. (2019) also suggested that nonallelic homologous recombination, i.e. unequal crossing-over between repeats, could contribute to increased CNVs, especially in regions with segmental duplications, although this was not the primary focus of their study. On the other hand, Bull *et al*. (2019) found that chronic exposure to Cu and/or Ni had no effect on rates of single nucleotide mutations, loss of heterozygosity events, or fitness suggesting that the CH lineage is mutationally robust. Overall, the observation by Chain, Flynn, *et al*. (2019) suggests that the rate of CNVs increased under exposure to a combination of Ni and Cu in the CH lineage, and our observation that absolute rDNA change rate in the SX lineage increased significantly under Ni exposure suggests that further examination of the impacts of exposure to these metals on recombination is warranted. Future studies should also estimate rates of mutation involving single nucleotide sites, deletions, and CNVs in the SX genomes and compare the results to those obtained for the CH lineage.

### rDNA sequence variation

We observed changes in haplotype distributions within lines over generations in both the SX and CH lineages. The SX lineage showed more consistent haplotype distributions across treatments, with haplotype 1 dominating in control and Cu40 treatments and haplotype 2 dominating in the Ni80 treatment. Moreover, the SX lineage did not show significant variation in expected heterozygosity with copy number changes. In contrast, there was a dynamic shift in haplotype dominance in the CH lineage over time, with haplotype 1 dominating across all treatments and haplotypes 2 and 4 becoming more prevalent in the control lines in generation 188. Moreover, the CH lineage exhibited a positive correlation between increased 28S copy number and expected heterozygosity, often due to the expansion of lower frequency haplotypes. This suggests that the distribution of haplotypes along the chromosomes was clustered in the progenitor female and recombination events often occurred in regions where the low-frequency haplotypes were located.

We would expect the fixation of haplotypes within rDNA arrays (concerted evolution) to occur more quickly when no new haplotypes are introduced by sexual reproduction. Indeed, some lines in the SX lineage, such as SX007, appear to be nearly fixed for a single haplotype, although very low-frequency variants might still exist. Other lines, like SX173, SX260, SX300, and SX395, also approached fixation. However, recombination can both drive and slow concerted evolution: although it can spread new haplotypes throughout an rDNA array, it can also create new variants through crossovers between different haplotypes. Thus, while the mechanisms of concerted evolution can maintain rDNA homogeneity, they can also slow the path to fixation of a single haplotype.

Ganley and Kobayashi (2011) suggested that rapid fixation is facilitated by frequent large deletions that remove significant portions of rDNA, creating a bottleneck effect. These deletions accelerate the homogenization process by reducing the number of rDNA repeats and increasing the likelihood that surviving haplotypes become dominant through subsequent recopying and amplification. This effect would be enhanced if haplotypes tend to be clustered within rDNA arrays, as Ganley and Kobayashi (2011) observed in *S. cerevisiae*, and we suggested here and in our study of rDNA variation in natural populations of *D. pulex* (Elguweidi and Crease 2024). Thus, large deletions play a key role in shaping the uniformity and efficiency of concerted evolution in rDNA arrays (Ganley and Kobayashi 2011). On the other hand, duplication of an rDNA segment containing a single haplotype could also help accelerate homogenization if the probability that recombination events occur within a haplotype cluster increases as the size of the cluster increases. Indeed, we observed that some individuals from natural populations of *D. pulex* with very high rDNA copy number were nearly fixed for a single haplotype (Elguweidi and Crease 2024), while individuals with substantially lower copy number contained multiple haplotypes at intermediate frequency.

## Conclusions

Our analysis of short-read whole-genome sequences from MA lines revealed a remarkable extent of rDNA change occurring in about 200 generations, comparable to the variation observed among individuals from natural sexual populations. This highlights the significant amount of rDNA variability that can arise without sexual reproduction in apomictically propagated lineages in the absence of selection. On the other hand, our analysis did not reveal a direct significant impact of Cu or Ni exposure on rDNA copy number or sequence variation in MA lines. However, metal exposure could influence rDNA copy number and sequence variation through changes in homologous recombination rates. Therefore, further research into the effects of metals on copy number change rate across various genomic regions is warranted. Our results also emphasize the importance of considering interval length when assessing rDNA copy number changes. Shorter intervals offer a more detailed view of the magnitude and frequency of rDNA copy number changes within lines, which may not be apparent over longer intervals. This approach would also make it easier to detect differences between the control and the metal-exposed lines, should they occur. Furthermore, the differential expansion of rDNA haplotypes suggests that they are likely clustered within chromosomes. Future research should focus on the functional significance of rDNA variation, including comparative studies across populations or species to identify correlations with traits like growth rate, reproductive success, or stress resistance. In addition, refining methodologies for accurate quantification of rDNA CNV is crucial for understanding how rDNA copy number and sequence vary in response to environmental stressors.

## Supplementary Material

jkae305_Supplementary_Data

## Data Availability

Genome sequence data can be found in the GenBank BioProject Database at https://www.ncbi.nlm.nih.gov/genbank/, with the corresponding accession numbers listed in Supplementary Table 1 in Supplementary File 2. The scripts and codes used for our analyses are available on GitHub at https://github.com/elguweidi/Daphnia.pulex.natural.population.git. Supplemental material available at G3 online.
