# Supplementary material for: Effect of copper and nickel exposure on ribosomal DNA variation in Daphnia pulex mutation accumulation lines
Source: G3 (Bethesda). 2024 Dec 23;15(3):jkae305. doi: 10.1093/g3journal/jkae305 (PMC11917474; doi:10.1093/g3journal/jkae305)
Supplement: jkae305_Supplementary_Data [file jkae305_supplementary_data.zip › File_S3_G3-2024-405610.pdf]

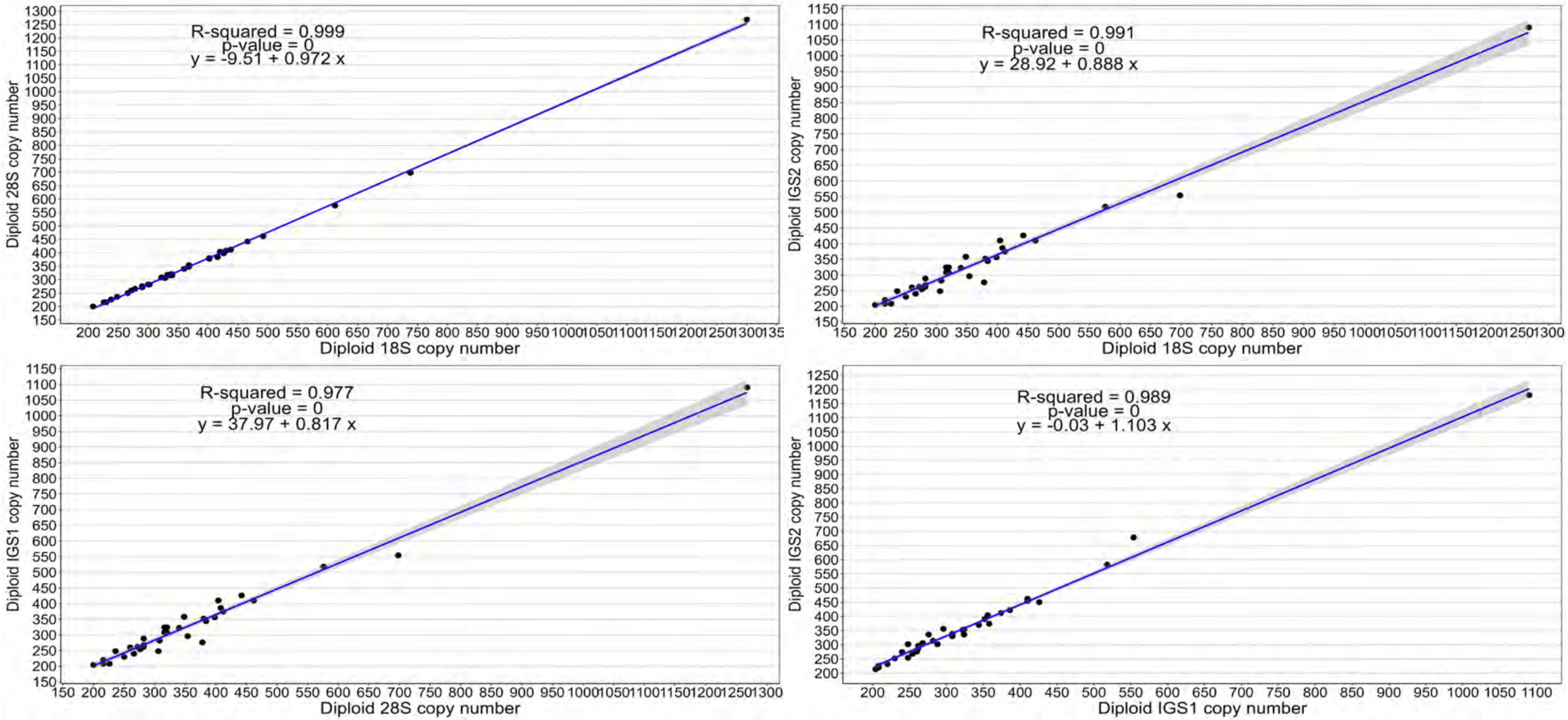

Figure S1. Regression of diploid copy number of rDNA regions in the SX MA lineage. The expected slope of the line is 1 if there is one copy of each region per rDNA repeat unit.

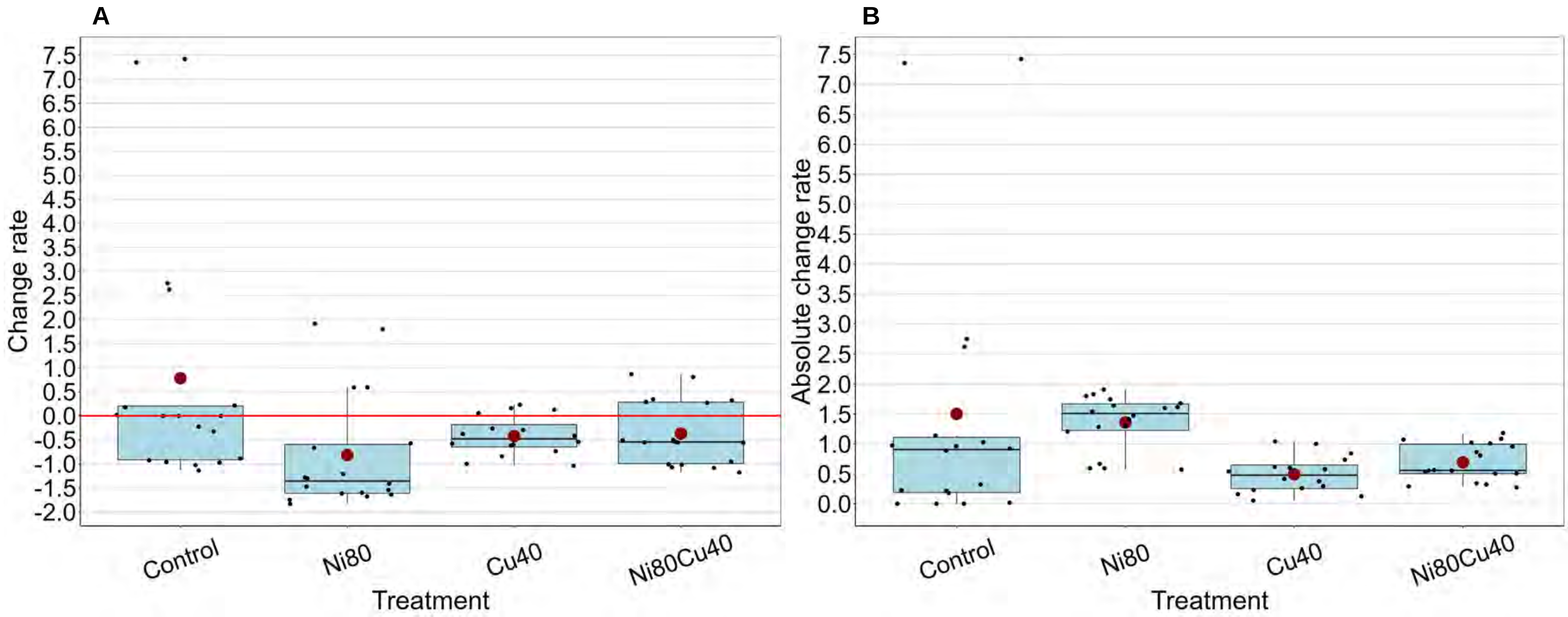

Figure S2. Rate of diploid 28S copy number change per generation (change rate) in the SX MA lines exposed to heavy metal. The diploid 28S copy number of the SX progenitor was estimated to be 378 based on the median value for the 9 control samples. All estimates are change rate from generation 0 to the sampling generation. The dark red point is the mean change rate. The box represents the Interquartile Range (IQR), the vertical lines represent 1.5*IQR, and the horizontal line in the box is the median. **A**. Change rate. The red line indicates the expected mean of 0 if there is no bias in direction of change. **B**. Absolute value of the change rate.

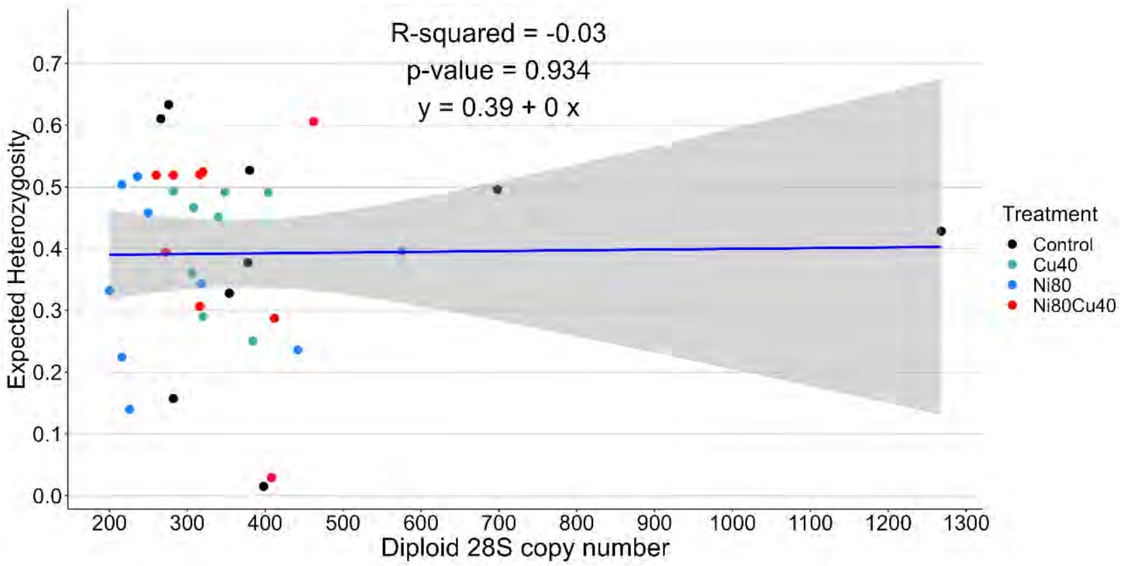

Figure S3. Regression analysis of the relationship between the diploid 28S copy number and the expected heterozygosity in 35 samples of the SX mutation accumulation lineage.

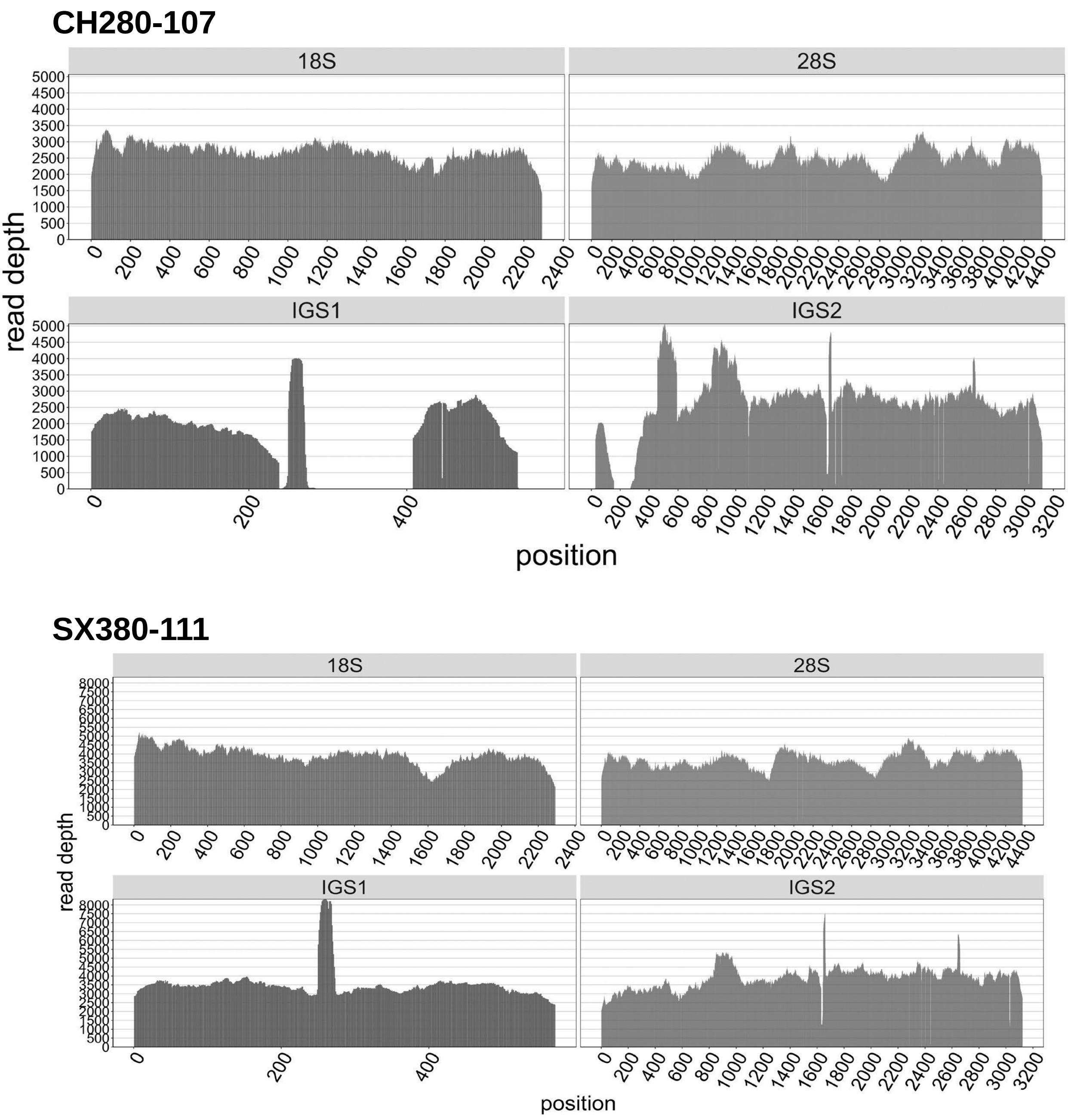

Figure S4. Depth of sequence reads across rDNA regions in one sample from each MA lineage. There is a ‘dead zone’ of no reads between nt 276 and nt 406 of the 577 nt IGS1 reference sequence in all CH samples. This dead zone does not occur in the SX samples.

A. CH025-87 **B. CH167-130**

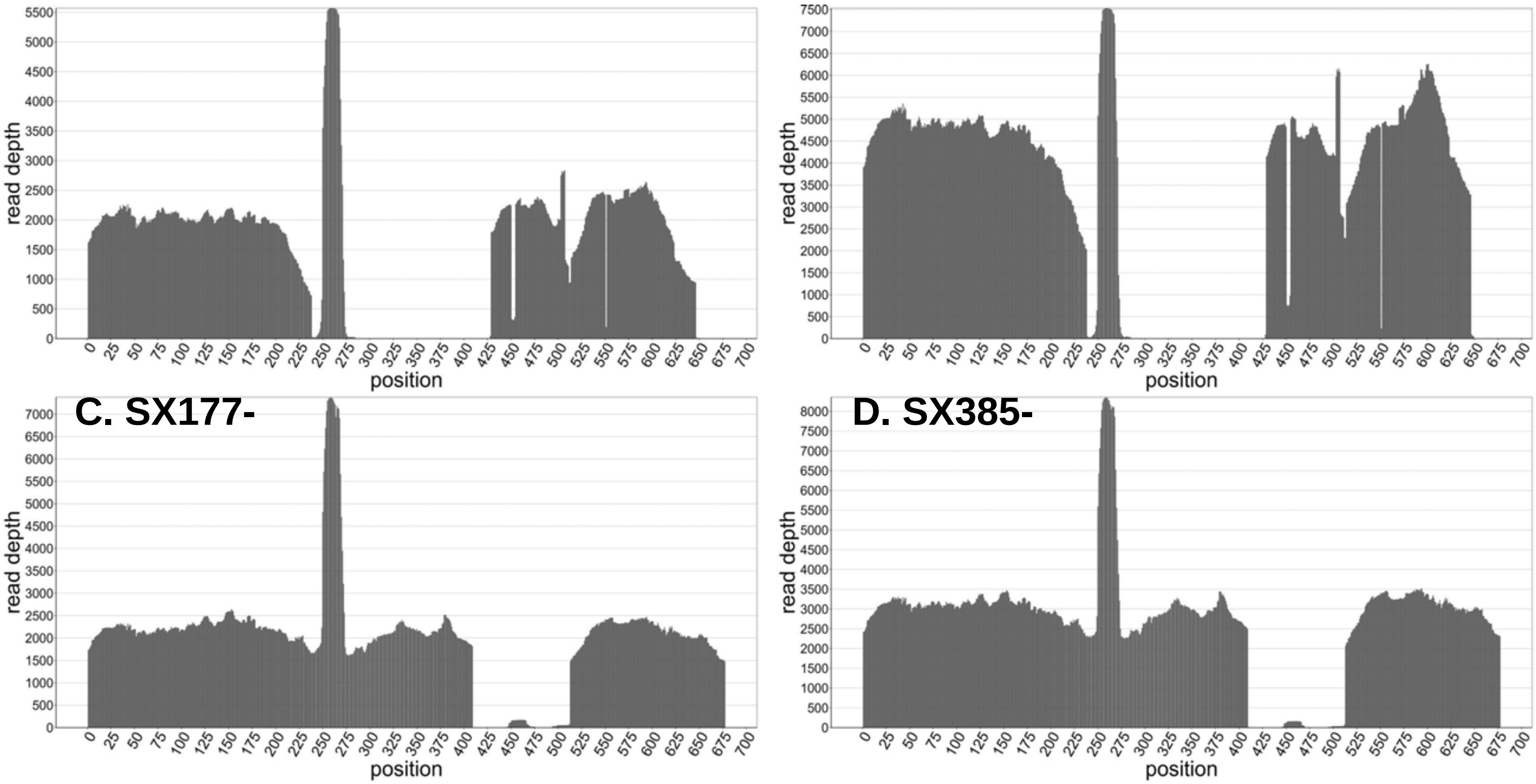

Figure S5. Read depth across an IGS1 reference sequence containing a *D. pulicaria*-specific 107 nt insert in two samples from each of the CH (A, B) and SX (C, D) lineages of *D. pulex*. We attributed the ‘dead zone’ identified in IGS1 of the CH samples (Figure S4) to the possibility that it is due to the hybrid nature of the CH lineage. Indeed, Ambrose and Crease (2011) observed that IGS sequences from *D. pulex* natural populations are very similar to each other and form a distinct cluster from IGS sequences from *D. pulicaria*. In addition, the *D. pulicaria* IGS1 has a 107-nt insertion located between nt 406 and 407 of our 571-nt reference sequence, immediately downstream of the "dead zone". Thus, this "dead zone" may be an artifact of the mapping software struggling to map sequence reads that span the *D. pulicaria*-specific insertion, resulting in erroneous estimates of read depth across IGS1. To test this, we realigned some genomes from the CH lineage with a reference sequence that includes the insertion and found that reads were mapped to the insert but not to the dead zone . We also mapped several SX genomes to the longer IGS sequence and found that a very small number of reads aligned with the center of the 107-nt insert but overall, the insert was absent in SX. This indicates that the mapping software effectively handles sequences even when a short deletion is present and suggests that the dead zone in CH IGS1 is due to a deletion in all rDNA repeats in the progenitor female's genome. In addition, all the CH rDNA repeats contain the *D. pulicaria*-specific insert at nt 406.

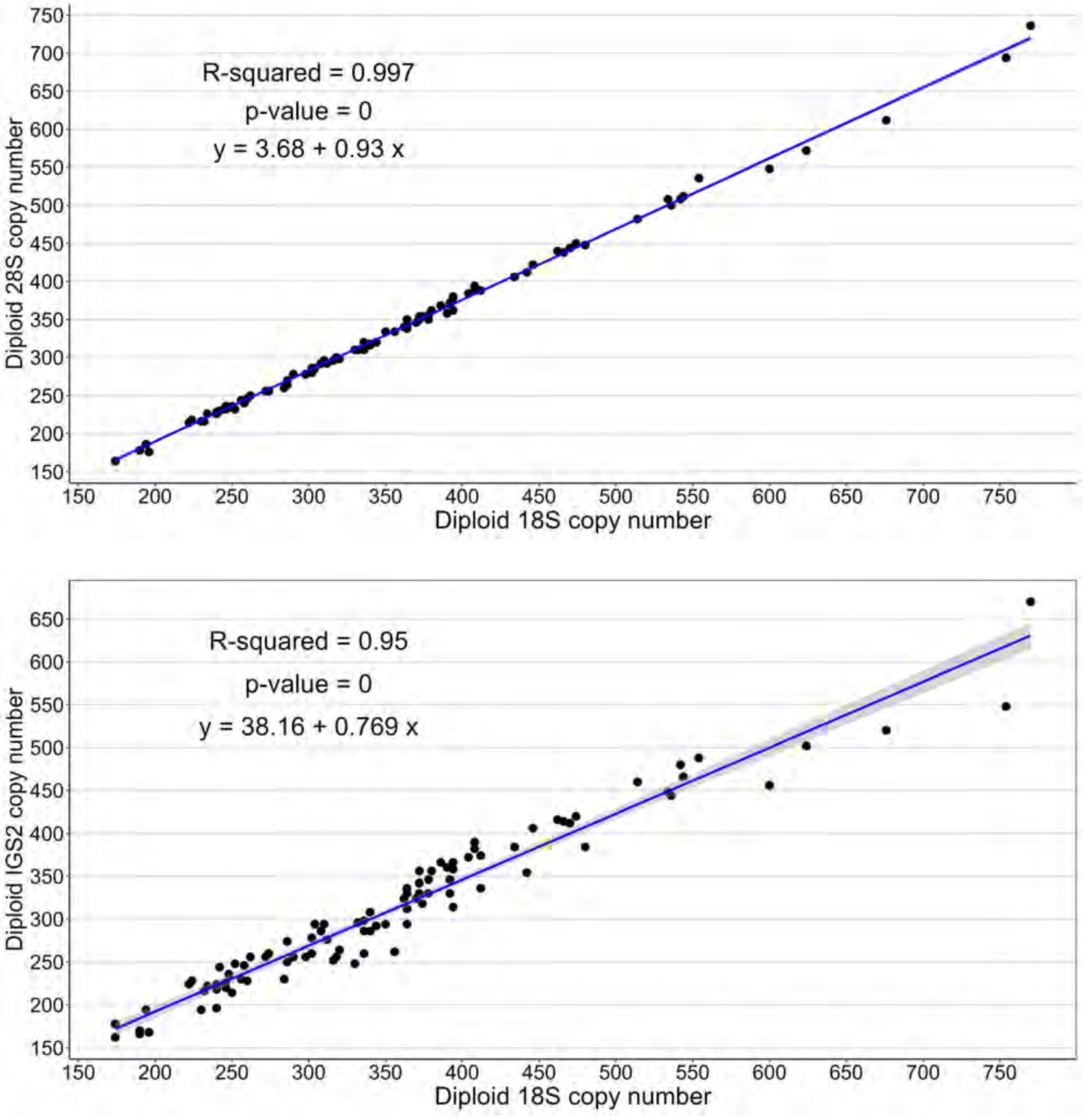

Figure S6. Regression of diploid copy number of rDNA regions in the CH MA lineage. The expected slope of the line is 1 if there is one copy of each region per rDNA repeat unit.

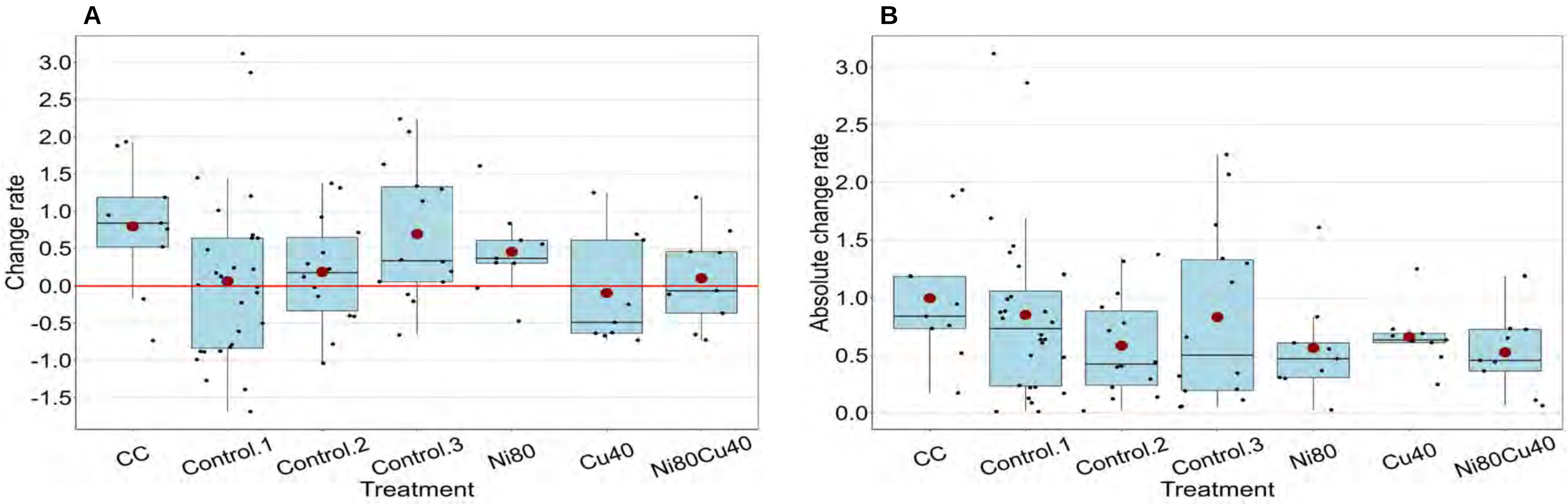

Figure S7. Rate of diploid 28S copy number change per generation (change rate) in the CH MA lines exposed to heavy metal. The diploid 28S copy number of the CH progenitor was estimated to be 299 based on the median value for the 28 control samples taken at generation 83. All estimates are change rate from generation 0 to the sampling generation. Fourteen control lines were sampled at three times and these groups are plotted separately. The dark red point is the mean change rate. The box represents the Interquartile Range (IQR), the vertical lines represent 1.5*IQR, and the horizontal line in the box is the median. **A**. Change rate. The red line indicates the expected mean of 0 if there is no bias in direction of change. **B**. Absolute value of the change rate.

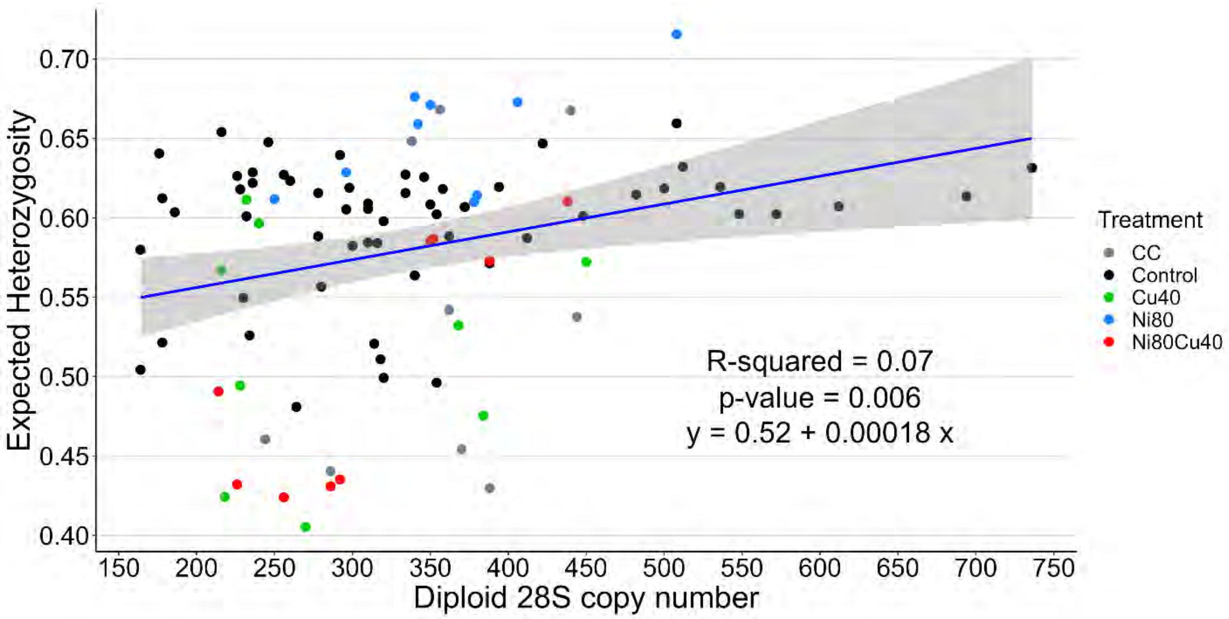

Figure S8. Regression analysis of the relationship between the diploid 28S copy number and the expected heterozygosity in 92 samples of the CH mutation accumulation lineage.

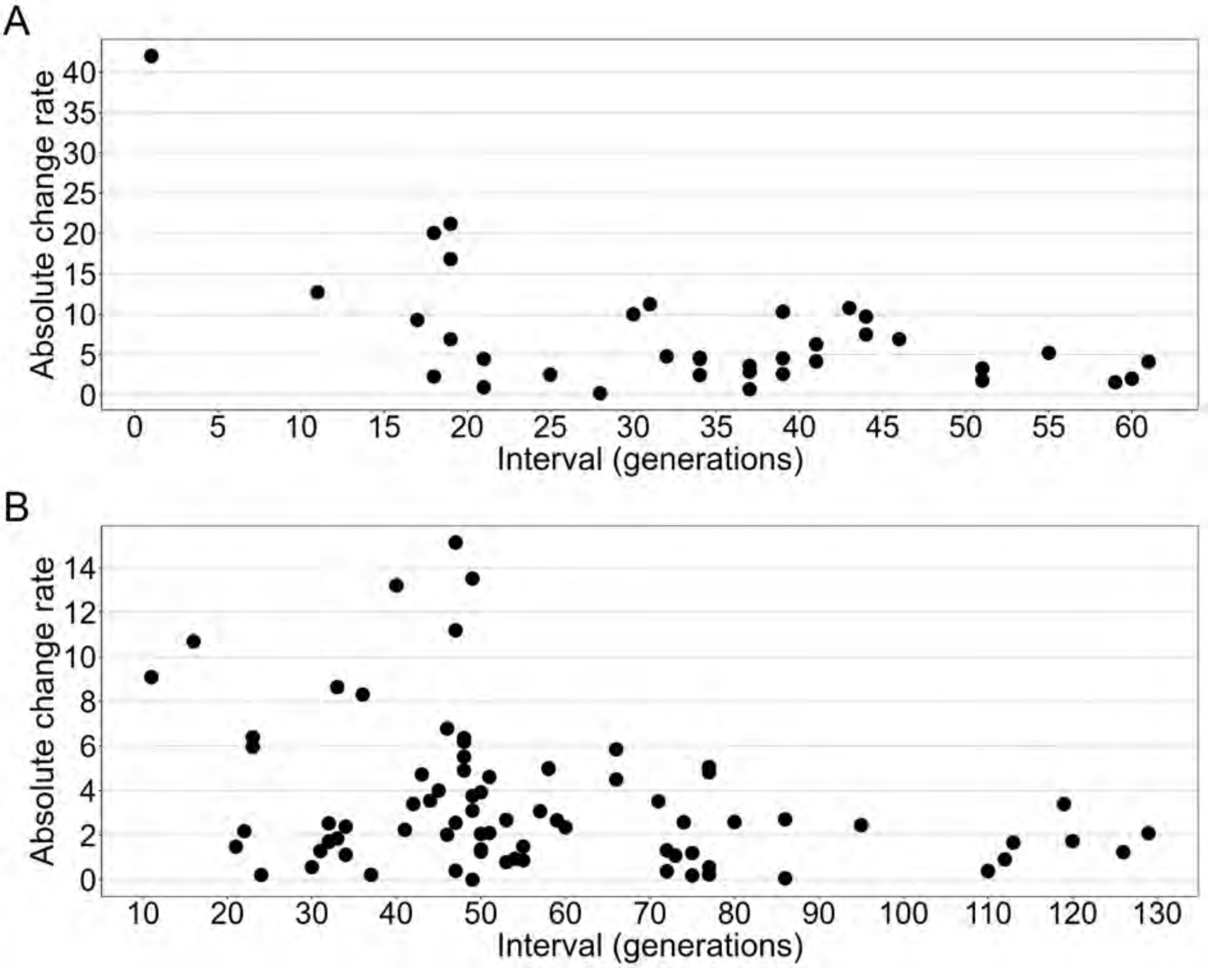

Figure S9. Absolute change rate of diploid 18S copy number in *D. pulex* MA lines as a function of the interval between samples. The data were taken from Harvey et al. (2020). 18S copy number was estimated using qPCR. Absolute change rate was estimated between all pairs of samples from lines that were sampled at least twice. **A.** SX lineage. **B**. CH lineage.
