# Supplementary material for: Effect of copper and nickel exposure on ribosomal DNA variation in Daphnia pulex mutation accumulation lines
Source: G3 (Bethesda). 2024 Dec 23;15(3):jkae305. doi: 10.1093/g3journal/jkae305 (PMC11917474; doi:10.1093/g3journal/jkae305)
Supplement: jkae305_Supplementary_Data [file jkae305_supplementary_data.zip › Supplemental_Material_Legends_G3-2024-405610.pdf]

Supplementary Files

FileS1.txt. *Daphnia pulex* reference sequences used to estimate rDNA copy number

FileS2.xlsx. Supplementary Tables

Table S1. rDNA variation in 127 samples of Daphnia pulex from mutation accumulation lines and a competitive control.

Table S2. Summary statistics for Diploid copy number in four rDNA regions in MA lines of *D. pulex*.

Table S3. Effects of metal exposure on diploid 28S copy number.

Table S4. Change in diploid 28S copy number per generation in the 14 CH control lines sampled at three time points.

Table S5. Comparison of diploid 18S copy number between this study and Harvey et al. (2020).

Table S6. Summary of 18S copy number and change rate for all lines sampled by Harvey et al. (2020) at generations 45, 70 and 100 in the CH and SX lineages.

FileS3.pdf. Supplementary Figures

Figure S1. Regression of diploid copy number of rDNA regions in the SX MA lineage.

Figure S2. Rate of diploid 28S copy number change per generation (change rate) in the SX MA lines exposed to heavy metal.

Figure S3. Regression analysis of the relationship between the diploid 28S copy number and the expected heterozygosity in 35 samples of the SX mutation accumulation lineage.

Figure S4. Depth of sequence reads across rDNA regions in one sample from each MA lineage.

Figure S5. Read depth across an IGS1 reference sequence containing a *D. pulicaria*-specific 107 nt insert in two samples from each of the CH (A, B) and SX (C, D) lineages of *D. pulex*.

Figure S6. Regression of diploid copy number of rDNA regions in the CH MA lineage.

Figure S7. Rate of diploid 28S copy number change per generation (change rate) in the CH MA lines exposed to heavy metal.

Figure S8. Regression analysis of the relationship between the diploid 28S copy number and the expected heterozygosity in 92 samples of the CH mutation accumulation lineage.

Figure S9. Absolute change rate of diploid 18S copy number in *D. pulex* MA lines as a function of the interval between samples.

FileS4.txt. rDNA haplotype sequences from two lineages (CH, SX) of *Daphnia pulex* mutation accumulation lines.
